# Locking of correlated neural activity to ongoing oscillations

**DOI:** 10.1371/journal.pcbi.1005534

**Published:** 2017-06-12

**Authors:** Tobias Kühn, Moritz Helias

**Affiliations:** 1 Institute of Neuroscience and Medicine (INM-6) and Institute for Advanced Simulation (IAS-6) and JARA BRAIN Institute I, Jülich Research Centre, Jülich, Germany; 2 Department of Physics, Faculty 1, RWTH Aachen University, Aachen, Germany; University of Pittsburgh, UNITED STATES

## Abstract

Population-wide oscillations are ubiquitously observed in mesoscopic signals of cortical activity. In these network states a global oscillatory cycle modulates the propensity of neurons to fire. Synchronous activation of neurons has been hypothesized to be a separate channel of signal processing information in the brain. A salient question is therefore if and how oscillations interact with spike synchrony and in how far these channels can be considered separate. Experiments indeed showed that correlated spiking co-modulates with the static firing rate and is also tightly locked to the phase of beta-oscillations. While the dependence of correlations on the mean rate is well understood in feed-forward networks, it remains unclear why and by which mechanisms correlations tightly lock to an oscillatory cycle. We here demonstrate that such correlated activation of pairs of neurons is qualitatively explained by periodically-driven random networks. We identify the mechanisms by which covariances depend on a driving periodic stimulus. Mean-field theory combined with linear response theory yields closed-form expressions for the cyclostationary mean activities and pairwise zero-time-lag covariances of binary recurrent random networks. Two distinct mechanisms cause time-dependent covariances: the modulation of the susceptibility of single neurons (via the external input and network feedback) and the time-varying variances of single unit activities. For some parameters, the effectively inhibitory recurrent feedback leads to resonant covariances even if mean activities show non-resonant behavior. Our analytical results open the question of time-modulated synchronous activity to a quantitative analysis.

## Author summary

In network theory, statistics are often considered to be stationary. While this assumption can be justified by experimental insights to some extent, it is often also made for reasons of simplicity. However, the time-dependence of statistical measures do matter in many cases. For example, time-dependent processes are examined for gene regulatory networks or networks of traders at stock markets. Periodically changing activity of remote brain areas is visible in the local field potential (LFP) and its influence on the spiking activity is currently debated in neuroscience. In experimental studies, however, it is often difficult to determine time-dependent statistics due to a lack of sufficient data representing the system at a certain time point. Theoretical studies, in contrast, allow the assessment of the time dependent statistics with arbitrary precision. We here extend the analysis of the correlation structure of a homogeneously connected EI-network consisting of binary model neurons to the case including a global sinusoidal input to the network. We show that the time-dependence of the covariances—to first order—can be explained analytically. We expose the mechanisms that modulate covariances in time and show how they are shaped by inhibitory recurrent network feedback and the low-pass characteristics of neurons. These generic properties carry over to more realistic neuron models.

## Introduction

To date it is unclear which channels the brain uses to represent and process information. A rate-based view is argued for by the apparent stochasticity of firing [1] and by the high sensitivity of the network dynamics to single spikes [2]. In an extreme view, correlated firing is a mere epiphenomenon of neurons being connected. Indeed, a large body of literature has elucidated how correlations relate to the connectivity structure [3–14]. But the matter is further complicated by the observation that firing rates and correlations tend to be co-modulated, as demonstrated experimentally and explained theoretically [4, 5]. If the brain employs correlated firing as a means to process or represent information, this requires in particular that the appearance of correlated events is modulated in a time-dependent manner. Indeed, such modulations have been experimentally observed in relation to the expectation of the animal to receive task-relevant information [15, 16] or in relation to attention [17].

Oscillations are an extreme case of a time-dependent modulation of the firing rate of cells. They are ubiquitously observed in diverse brain areas and typically involve the concerted activation of populations of neurons [18]. They can therefore conveniently be studied in the local field potential (LFP) that represents a complementary window to the spiking activity of individual neurons or small groups thereof: It is composed of the superposition of the activity of hundreds of thousands to millions of neurons [19, 20] and forward modeling studies have confirmed [21] that it is primarily driven by the synaptic inputs to the local network [22–24]. As the LFP is a quantity that can be measured relatively easily, this mesoscopic signal is experimentally well documented. Its interpretation is, however, still debated. For example, changes in the amplitude of one of the components of the spectrum of the LFP have been attributed to changes in behavior (cf. e.g. [25]).

**Fig 1:**
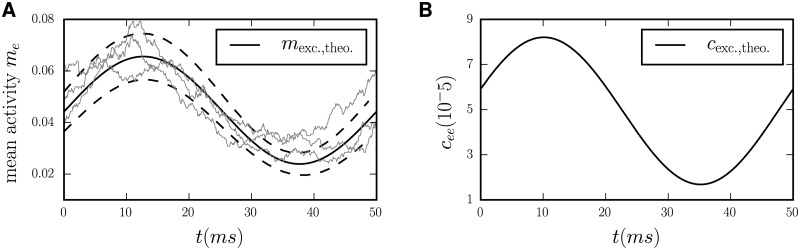
**A** Time-varying mean activity of the excitatory population $m_{E}(t)=N_{E}^{-1}\sum_{i\in E}\;n_{i}(t)$ in a balanced EI-network (parameters given in Table 1). Thin gray lines are the outcomes of three independent simulations, the solid black line indicates the mean activity predicted by the theory (Eqs (7) and (8)). Dashed black lines indicate the range of expected fluctuations of the population activity (± one standard deviation): The square of the fluctuation magnitude is given by the variance of the population activity $\frac{a_{E}}{N_{E}}+c_{EE}$ (Eqs (3) and (4)). **B** Population-averaged cross covariance $c_{EE}=\frac{1}{N_{E}(N_{E}-1)}\sum_{i\neq j\in E}c_{ij}$ .

A particular entanglement between rates and correlations is the correlated firing of spikes in pairs of neurons in relation to the phase of an ongoing oscillation. With the above interpretation of the LFP primarily reflecting the input to the cells, it is not surprising that the mean firing rate of neurons may modulate in relation to this cycle. The recurrent network model indeed confirms this expectation, as shown in Fig 1A. It is, however, unclear if and by which mechanisms the covariance of firing follows the oscillatory cycle. The simulation shown in Fig 1B indeed exhibits a modulation of the covariance between the activities of pairs of cells. Such modulations have also been observed in experiments:

Denker et al. [26] have shown that the synchronous activation of pairs of neurons within milliseconds preferentially appears at a certain phase of the oscillatory component of the LFP in the beta-range—in their words the spike-synchrony is “phase-locked” to the beta-range of the LFP. They explain their data by a conceptual model, in which an increase in the local input, assumed to dominate the LFP, leads to the activation of cell assemblies. The current work investigates an alternative hypothesis: We ask if a periodically-driven random network is sufficient to explain the time-dependent modulation of covariances between the activities of pairs of cells or whether additional structural features of the network are required to explain this experimental observation.

To investigate the mechanisms causing time-dependent covariances in an analytically tractable case, we here present the simplest model that we could come up with that captures the most important features: A local network receiving periodically changing external input. The randomly connected neurons receive sinusoidally modulated input, interpreted as originating from other brain areas and mimicking the major source of the experimentally observed LFP. While it is obvious that the mean activity in a network follows an imposed periodic stimulation, it is less so for covariances. In the following we will address the question why they are modulated in time as well. Extending the analysis of mean activities and covariances in the stationary state [13, 27, 28], we here expose the fundamental mechanisms that shape covariances in periodically driven networks.

Our network model includes five fundamental properties of neuronal dynamics: First, we assume that the state of low and irregular activity in the network [1] is a consequence of its operation in the balanced state [29, 30], where negative feedback dynamically stabilizes the activity. Second, we assume that each neuron receives a large number of synaptic inputs [31], each of which only has a minor effect on the activation of the receiving cell, so that total synaptic input currents are close to Gaussian. Third, we assume the neurons are activated in a threshold-like manner depending on their input. Fourth, we assume a characteristic time scale *τ* that measures the duration of the influence a presynaptic neuron has on its postsynaptic targets. Fifth, the output of the neuron is dichotomous or binary, spike or no spike, rather than continuous. As a consequence, the variance of the single unit activity is a direct function of its mean.

We here show how each of the five above-mentioned fundamental properties of neuronal networks shape and give rise to the mechanisms that cause time-dependent covariances. The presented analytical expressions for the linear response of covariances expose two different paths by which a time-dependence arises: By the modulation of single-unit variances and by the modulation of the linear gain resulting from the non-linearity of the neurons. The interplay of negative recurrent feedback and direct external drive can cause resonant behavior of covariances even if mean activities are non-resonant. Qualitatively, these results explain the modulation of synchrony in relation to oscillatory cycles that are observed in experiments, but a tight locking of synchronous events to a particular phase of the cycle is beyond the mechanisms found in the here-studied models.

## Results

To understand the locking of synchronous activity to an oscillatory cycle, as observed experimentally, we here need to consider time-dependent network states. We are in particular interested in the covariance between two stochastic variables *x*_1_ and *x*_2_, which is defined as *c*(*t*) = 〈*δx*_1_(*t*)*δx*_2_(*t*)〉 = 〈(*x*_1_(*t*) − 〈*x*_2_(*t*)〉) (*x*_1_(*t*) − 〈*x*_2_(*t*)〉〉, where 〈…〉 denotes the average over realizations. In words, the covariance in a time-dependent setting measures the co-variability of a pair of signals with respect to their respective mean. The mean value itself may depend on time. Only if this quantity can be determined with sufficient accuracy, time-dependent covariances can be calculated correctly. This is the source of the technical problems occurring in the context of a time-dependent covariance: It may be hard to assess the covariance, much more its time-dependence, because it is overshadowed by the time-varying mean. If a stochastic model is given, however, disentangling the time dependence of different cumulants, like mean and covariance, is possible. A theoretical study to understand the prevalent mechanisms that cause time-dependent covariances in a network model is therefore a necessary first step. In the following we identify these mechanisms by which time-dependent covariances of activities arise in oscillatory-driven recurrent networks. In Fig 1A we show the population-averaged activity of the excitatory population activity in a balanced EI-network together with the theoretical prediction to be developed in the sequel: The fluctuations around the mean show a wider spread close to the peak of the oscillation than at the trough. Correspondingly, the covariance between pairs of neurons in panel **B** has its peaks and troughs at points of high and low variability of the population activity in **A**, respectively.

### Binary network model and its mean field equations

**Fig 2:**
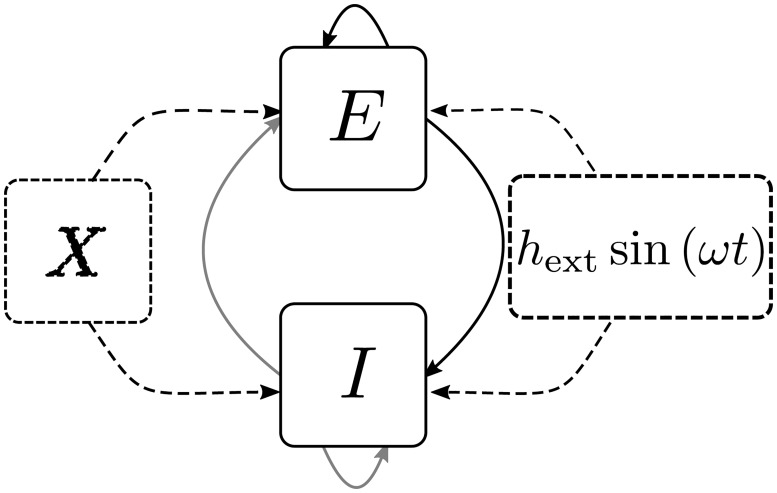
Recurrent balanced network driven by oscillatory input. Recurrently connected excitatory (*E*) and inhibitory (*I*) populations (Erdős-Rényi random network with connection probability *p*) receiving input from an external (*X*) excitatory population. Additionally, all neurons in the microcircuit receive a sinusoidal signal of amplitude *h*_ext_ and frequency *ω*, representing the oscillatory activity received from external brain areas.

To address our central question, whether a periodically-driven random network explains the experimental observations of time-modulated pairwise covariances, we consider a minimal model here. It consists of one inhibitory (*I*) population and, in the latter part of the paper, additionally one excitatory population (*E*) of binary model neurons [6, 27, 29, 32]. Neurons within these populations are recurrently and randomly connected. All neurons are driven by a global sinusoidal input mimicking the incoming oscillatory activity that is visible in the LFP, illustrated in Fig 2. The local network may in addition receive input from an external excitatory population (*X*), representing the surrounding of the local network. The fluctuations imprinted by the external population, providing shared inputs to pairs of cells, in addition drive the pairwise covariances within the network [13, c.f. especially the discussion]. Therefore we need the external population *X* to arrive at a realistic setting that includes all sources of covariances. In the following, we extend the analysis of cumulants in networks of binary neurons presented in [6, 13, 27, 28, 33] to the time-dependent setting. This formal analysis allows us to obtain analytical approximations for the experimentally observable quantities, such as pairwise covariances, that expose the mechanisms shaping correlated network activity.

Binary model neurons at each point in time are either inactive *n*_*i*_ = 0 or active *n*_*i*_ = 1. The time evolution of the network follows the Glauber dynamics [34]; the neurons are updated asynchronously. At every infinitesimal time step *dt*, any neuron is chosen with probability $\frac{dt}{\tau }$. After an update, neuron *i* is in the state 1 with the probability *F*_*i*_(***n***) and in the 0-state with probability 1 − *F*_*i*_(***n***), where the activation function *F* is chosen to be

$$\begin{array}{cc} F_{i}\left(n\right) & =H\left(h_{i}-\theta_{i}\right)\\ h_{i} & =\sum_{k=1}^{N}J_{ik}n_{k}+h_{\text{ext}}\text{sin}\left(\omega t\right)+\xi_{i}\\ H(x) & =\left\{\begin{array}{cc} 1 & \text{if}\;x\geq 0\\ 0 & \text{if}\;x<0 \end{array}\right.. \end{array}$$ (1)

We here introduced the connectivity matrix *J* with the synaptic weights $J_{ij}\in \mathbb{R}$ describing the influence of neuron *j* on neuron *i*. The weight *J*_*ij*_ is negative for an inhibitory neuron *j* and positive for an excitatory neuron. Due to the synaptic coupling the outcome of the update of neuron *i* potentially depends on the state ***n*** = (*n*_1_, …, *n*_*N*_) of all other neurons in the network. Compared to the equations in [13, page 4], we added an external sinusoidal input to the neurons representing the influence of other cortical or subcortical areas and Gaussian uncorrelated noise with vanishing mean 〈*ξ*_*i*_〉 = 0 and covariance $\langle \xi_{i}\xi_{j}\rangle =\delta_{ij}\sigma_{\text{noise}}^{2}$. The threshold *θ*_*i*_ depends on the neuron type and will be chosen according to the desired mean activity.

We employ the neural simulation package NEST [35, 36] for simulations. Analytical results are obtained by mean-field theory [6, 13, 27, 28, 37, 38] and are described for completeness and consistency of notation in the section “*Methods*”. In the main text we only mention the main steps and assumptions entering the approximations. The basic idea is to describe the time evolution of the Markov system in terms of its probability distribution *p*(***n***, *t*). Using the master Eq 14 we obtain ordinary differential equations (ODEs) for the moments of *p*(***n***, *t*). In particular we are interested in the population averaged mean activities *m*_*α*_, variances *a*_*α*_, and covariances *c*_*αβ*_

$$\begin{array}{ccc} m_{\alpha }\left(t\right) & ≔ & \frac{1}{N_{\alpha }}\sum_{i\in \alpha }\left\langle n_{i}\left(t\right)\right\rangle \end{array}$$ (2)

$$\begin{array}{ccc} a_{\alpha }\left(t\right) & ≔ & \frac{1}{N_{\alpha }}\sum_{i\in \alpha }\left\langle n_{i}\left(t\right)\right\rangle -{\left\langle n_{i}\left(t\right)\right\rangle }^{2} \end{array}$$ (3)

$$\begin{array}{ccc} c_{\alpha \beta }\left(t\right) & ≔ & \frac{1}{N_{\alpha }N_{\beta }}\sum_{i\in \alpha ,j\in \beta ,i\neq j}\left\langle n_{i}\left(t\right)n_{j}\left(t\right)\right\rangle -\left\langle n_{i}\left(t\right)\right\rangle \left\langle n_{j}\left(t\right)\right\rangle , \end{array}$$ (4)

which are defined as expectation values 〈〉 over realizations of the network activity, where the stochastic update of the neurons and the external noisy input presents the source of randomness in the network. The dynamics couples moments of arbitrarily high order [33]. To close this set of equations, we neglect cumulants of order higher than two, which also approximates the input by a Gaussian stochastic variable with cumulants that vanish for orders higher than two [39]. This simplification can be justified by noticing that the number of neurons contributing to the input is large and their activity is weakly correlated, which makes the central limit theorem applicable. In a homogeneous random network, on expectation there are *K*_*αβ*_ = *p*_*αβ*_ *N*_*β*_ synapses from population *β* to a neuron in population *α*. Here *p*_*αβ*_ is the connection probability; the probability that there is a synapse from any neuron in population *β* to a particular neuron in population *α* and *N*_*α*_ is the size of the population. Mean Eq (2) and covariance Eq (4) then follow the coupled set of ordinary differential equations (ODEs, see section II A in S1 Text for derivation)

$$\begin{array}{ccc} \tau \frac{d}{dt}m_{\alpha }\left(t\right) & = & -m_{\alpha }\left(t\right)+\varphi \left(\mu_{\alpha }\left(m\left(t\right),h_{\text{ext}}\text{sin}\left(\omega t\right)\right),\sigma {}_{\alpha }\left(m\left(t\right),c\left(t\right)\right)\right) \end{array}$$ (5)

$$\begin{array}{ccc} \tau \frac{d}{dt}c_{\alpha \beta }\left(t\right) & = & \{-c_{\alpha \beta }\left(t\right)+\sum_{\gamma }[S\left(\mu_{\alpha }\left(m\left(t\right),h_{\text{ext}}\text{sin}\left(\omega t\right)\right),\sigma_{\alpha }\left(m\left(t\right),c\left(t\right)\right)\right)\\ & & \times K_{\alpha \gamma }J_{\alpha \gamma }\;\left(c_{\gamma \beta }\left(t\right)+\delta_{\gamma \beta }\frac{a_{\beta }\left(t\right)}{N_{\beta }}\right)]\}+\left\{\alpha ↔\beta \right\}, \end{array}$$ (6)

where *α* ↔ *β* indicates the transposed term. The Gaussian truncation employed here is parameterized by the mean *μ*_*α*_ and the variance $\sigma_{\alpha }^{2}$ of the summed input to a neuron in population *α*. These, in turn, are functions of the mean activity and the covariance, given by Eqs (18) and (19), respectively.

Here *φ* is the expectation value of the activation function, which is smooth, even though the activation function itself is a step function, therefore not even continuous. The function *φ* fulfills lim_***m*** → 0_ *φ* = 0 and lim_***m*** → 1_ *φ* = 1 and monotonically increases. Its derivative *S* with respect to *μ* has a single maximum and is largest for the mean input *μ* within a region with size *σ* around the threshold *θ*. *S* measures the strength of the response to a slow input and is therefore termed susceptibility. The definitions are given in “Methods” in Eqs (17) and (20).

The stationary solution (indicated by a bar) of the ODEs Eqs (5) and (6) can be found by solving the equations

$$\begin{array}{ccc} \bar{m} & = & \varphi \left(\bar{m}\right) \end{array}$$ (7)

$$\begin{array}{ccc} 2\bar{c} & = & SKJ\;\left(\bar{c}+\frac{\bar{a}}{N}\right)+\text{transposed} \end{array}$$ (8)

numerically and self-consistently, as it was done in [13, 27, 33].

The full time-dependent solution of Eqs (5) and (6) can, of course, be determined numerically without any further assumptions. Besides the comparison with simulation results, this will give us a check for the subsequently applied linear perturbation theory. The resulting analytical results allow the identification of the major mechanisms shaping the time-dependence of the first two cumulants. To this end, we linearize the ODEs Eqs (5) and (6) around their stationary solutions. We only keep the linear term of order *h*_ext_ of the deviation, justifying a Fourier ansatz for the solutions. For the mean activities this results in $m_{\alpha }\left(t\right)={\bar{m}}_{\alpha }+\delta m_{\alpha }\left(t\right)={\bar{m}}_{\alpha }+M_{\alpha }^{1}e^{i\omega t}$ with

$$\begin{array}{c} M_{\alpha }^{1}=\sum_{\beta }U_{\alpha \beta }M_{\beta }^{1}=\sum_{\beta }U_{\alpha \beta }\frac{h_{\text{ext}}{\left(U^{-1}S\left(\bar{\mu },\bar{\sigma }\right)\right)}_{\beta }\left(-i\tau \omega +1-\lambda_{\beta }\right)}{{\left(\tau \omega \right)}^{2}+{\left(1-\lambda_{\beta }\right)}^{2}}. \end{array}$$ (9)

**Fig 3:**
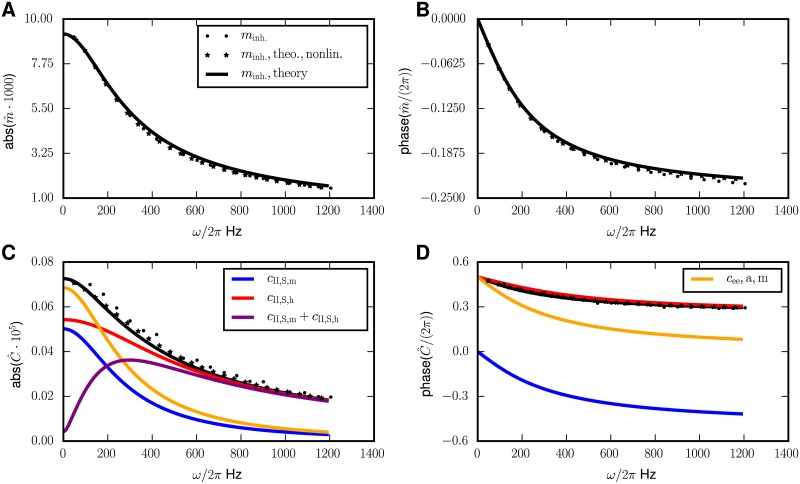
Periodically driven single population network. Dependence of the modulations of the mean activity and covariances on the driving frequency *ω*. **A** Amplitude of modulation of mean activity. **B** Phase of modulation of mean activity relative to the external drive. **C** Amplitude of modulation of covariances. **D** Phase of modulation of covariance relative to the external drive. In all panels, the analytical predictions (Eqs (9) and (39)) are shown as solid black curves. The black curve is the complete solution. The different contributions to the time-dependent covariances, identified in Eq (12), are shown separately: The *S*_*h*_-term in red, the *S*_*m*_-term in blue, their sum in purple, and the *a*-term in orange. Numerical solutions of the full mean-field equations (Eqs (5) and (6)) are shown as stars and simulation results by dots (only indicated in the legend of A). The numerical results are obtained by using the integrate.ode-method from the python-package scipy [41] with the option “lsoda”, meaning that either implicit Adams- or backward differentiation-algorithms (depending on the given problem) are used. Network parameters: Number of neurons *N*_*I*_ = 5000, connection probability *p*_*II*_ = 0.1, coupling strength *J*_*II*_ = −1, mean activity *m*_*I*_ ≈ 0.3, and $\sigma_{\text{noise}}=\sigma_{\text{system}}\;≔\;\sqrt{J_{II}^{2}p_{II}N_{I}m_{I}\left(1-m_{I}\right)}\approx 10.2$.

The time-dependence of *σ* was neglected here, which can be justified for large networks (“*Methods*”, Eqs (22) and (30)). The matrix *U* represents the basis change that transforms ${\bar{W}}_{\alpha \beta }\;≔\;S\left({\bar{\mu }}_{\alpha },{\bar{\sigma }}_{\alpha }\right)\;K_{\alpha \beta }J_{\alpha \beta }$ into a diagonal matrix with λ_*α*_ the corresponding eigenvalues. We see that, independent of the number of populations or the detailed form of the connectivity matrix, the amplitude of the time-dependent part of the mean activities has the shape of a low-pass-filtered signal to first order in *h*_ext_. Therefore the phase of *δ**m*** lags behind the external drive and its amplitude decreases asymptotically like $\frac{1}{\omega }$, as can be seen in Fig 3A and 3B.

If we also separate the covariances into their stationary part and a small deviation that is linear in the external drive, $c_{\alpha \beta }\;\left(t\right)={\bar{c}}_{\alpha \beta }+\delta c_{\alpha \beta }\;\left(t\right)$, expand *S* (*μ*_*α*_ (*t*), *σ*_*α*_ (*t*)) and *a* (*t*) around their stationary values, keeping only the terms of order *h*_ext_ and neglect contributions from the time-dependent variation of the variance of the input *σ*^2^ (see “Methods”, especially Eq (30) for a discussion of this point), we get the ODE

$$\begin{array}{ccc} & & \tau \frac{d}{dt}\delta c\left(t\right)+2\delta c\left(t\right)-\bar{W}\delta c\left(t\right)-{\left(\bar{W}\delta c\left(t\right)\right)}^{T}\\ & = & \{\underset{\text{modulated-autocorrelations-drive}}{\underset{︸}{\bar{W}\;\text{diag}\left(\frac{1-2\bar{m}}{N}\right)\text{diag}\left(\delta m\left(t\right)\right)}}\\ & + & \left[\underset{\text{recurrent}\;\text{drive}}{\underset{︸}{\text{diag}\left(K\;⊛\;J\;\delta m\left(t\right)\right)}}+\underset{\text{direct}\;\text{drive}}{\underset{︸}{h_{\text{ext}}\text{sin}\left(\omega t\right)}}\right]\;\text{diag}\left(\frac{\partial S}{\partial \mu \;\left(t\right)}\right)\;K\;⊛\;J\;{\bar{c}}^{\text{total}}\}\\ & + & {\left\{...\right\}}^{T}, \end{array}$$ (10)

where we introduced the point-wise (Hadamard) product ⊛ of two matrices *A* and *B* [see 40, for a consistent notation of matrix operations] as (*A* ⊛ *B*)_*ij*_ ≔ *A_ij_B_ij_*, defined the matrix with the entries diag (***x***)_*ij*_ := *δ*_*ij*_ *x*_*i*_ for the vector ***x*** = (*x*_1_, ‥, *x*_*n*_) and set ${\bar{c}}^{total}\;≔\;\bar{c}+diag\left(\frac{\bar{a}}{N}\right)$ to bring our main equation into a compact form.

We can now answer the question posed in the beginning: Why does a global periodic drive influence the cross covariances in the network at all and does not just make the mean activities oscillate? First, the variances are modulated with time, simply because they are determined via Eq (3) by the modulated mean activities. A neuron *i* with modulated autocorrelation *a*_*i*_(*t*) projects via *J*_*ji*_ to another neuron *j* and therefore shapes the pairwise correlation *c*_*ji*_(*t*) in a time-dependent way. We call this effect the “modulated-autocovariances-drive”, indicated by the curly brace in the second line of Eq (10). Its form in index notation is $\left[\bar{W}\;diag\left((1-2\bar{m})/N\right)diag\left(\delta m\left(t\right)\right){]}_{\alpha \beta }={\bar{W}}_{\alpha \beta }\;(1-2{\bar{m}}_{\beta })/N_{\beta }\;\delta m_{\beta }(t\right)$. This is the low-pass-filtered input.

The other contributions are a bit more subtle and less obvious, as they are absent in networks with a linear activation function. The derivative of the expectation value of the activation function, the susceptibility, contributes linearly to the ODE of the covariances. As the threshold-like activation function gives rise to a nonlinear dependence of *φ* on the mean input *μ*, the susceptibility *S* = *φ*′ is not constant, but depends on the instantaneous mean input. The latter changes as a function of time by the direct external drive and by the recurrent feedback of the oscillating mean activity, indicated by the terms denoted by the curly braces in the third line of Eq (10). Together, we call these two term the “susceptibility terms”. Both terms are of the same form

$$\begin{array}{c} {\left[\text{diag}\left(\delta \mu (t)\right)\text{diag}\left(\frac{\partial S}{\partial \mu \left(t\right)}\right)\;K\;⊛\;J\;{\bar{c}}^{\text{total}}\right]}_{\alpha \beta }=\delta \mu_{\alpha }\left(t\right)\;\frac{\partial S_{\alpha }}{\partial \mu_{\alpha }}\;\sum_{\gamma }\;K_{\alpha \gamma }J_{\alpha \gamma }\;\left({\bar{c}}_{\gamma \beta }+\delta_{\gamma \beta }\frac{{\bar{a}}_{\beta }}{N_{\beta }}\right), \end{array}$$ (11)

but with different *δμ*_*α*_. This form shows how the time-dependent modulation of the mean input *δμ*_*α*_, by the second derivative of the gain function $\frac{\partial S_{\alpha }}{\partial \mu_{\alpha }}=\varphi^{\prime \prime }$, influences the transmission of covariances. The sum following $\frac{\partial S_{\alpha }}{\partial \mu_{\alpha }}$ is identical to the one in the static case Eq (8). For the “recurrent drive”, the time-dependent input is given by *δμ*_*α*_(*t*) = ∑_*β*_ *K*_*αβ*_ *J*_*αβ*_ *δm*_*β*_(*t*), which is a superposition of the time-dependent activities that project to population *α* and is therefore low-pass-filtered, too. The term due to “direct drive” is *δμ*_*α*_(*t*) = *h*_ext_ sin(*ωt*).

We solve Eq (10) by transforming into the eigensystem of $\bar{W}$ and inserting a Fourier ansatz, $\delta c_{\alpha \beta }\left(t\right)=C_{\alpha \beta }^{1}e^{i\omega t}$. The solution consists of a low-pass filtered part coming from the direct drive and two parts that are low-pass filtered twice, coming from the recurrent drive and the modulated-autocovariances-drive. For a detailed derivation, consult the section “*Covariances: Stationary part and response to a perturbation in linear order*”.

We have calculated higher Fourier modes of the simulated network activity and of the numerical solution of the mean-field equations to check if they are small enough to be neglected, so that the response is dominated by the linear part. Of course, it would be possible to derive analytical expressions for those as well. However, we will see that the linear order and the corresponding first harmonic qualitatively and for remarkably large perturbations even quantitatively gives the right predictions. The limits of this approximation are analyzed in Fig D in S1 Text. We will therefore constrain our analysis to controlling the higher harmonics through the numerical solution.

In the following we will study three different models of balanced neuronal networks to expose the different mechanisms in their respective simplest setting.

#### Single population

As a first example, we quantitatively study the particular case of a single population, which has to be inhibitory to ensure stable stationary activity. Let us look at the behavior of the different contributions in Eq (10) to the modulated covariance and their mutual relation. Written explicitly, the terms driving the time variation of the covariance are

$$\begin{array}{ll} & \left(\overset{\text{susceptibility terms};\text{partly cancel}}{\overset{︷}{\underset{S_{m}\text{-term }\propto \frac{1}{\omega }\text{ for big }\omega }{\underset{︸}{K\;J\;\delta m\left(t\right)}}+\underset{S_{h}\text{-term does not scale with }\omega }{\underset{︸}{h_{\text{ext}}\text{ sin}\left(\omega t\right)}}}}\right)\frac{\partial S}{\partial \mu }K\;J\underset{\text{partly cancel}}{\underset{︸}{\left(\bar{c}+\frac{\bar{a}}{N}\right)}}\\ + & \underset{a\text{-term }\propto \frac{1}{\omega }\text{ for big }\omega }{\underset{︸}{\bar{W}\left(1-2\bar{m}\right)\frac{\delta m\left(t\right)}{N}}}. \end{array}$$ (12)

With respect to their dependence on the number of synaptic connections |*K*|, the sum of the two susceptibility terms is of the same order of magnitude as the modulated-autocovariances-drive (cf. 37 in the section “*Methods*”), therefore their interplay determines the shape of the solution of Eq (10) and we cannot neglect either term in favor of the other.

To analyze the contributions to *δc*, it is reasonable to first focus on the quasi-static case *ω* → 0, because its analysis is simplest and, due to the continuity of the observed quantities, it carries over to the case of biologically relevant small frequencies up to the *β*-range. For *ω* → 0, the solution *δc* in Eq (10) has the same sign as the sum of the inhomogeneities, because it is given by a multiplication with 0.5 (1 − *W*)^−1^, where *W* < 0. The main information—especially about the sign—is therefore already included in these inhomogeneities, that we termed “recurrent drive” and “direct drive” (the susceptibility terms) and “modulated-autocovariances-drive” in the previous section. The modulation of the covariance *δc*(*t*) then results by low pass filtering their sum. Individually they yield the *S*_*m*_-term and *S*_*h*_-term (together the *S*-terms) and the *a*-term, respectively.

In a general balanced network, the deviation of the mean activity from the stationary solution *δ**m***(*t*) is in phase with the perturbation for *ω* ≈ 0 and lags behind it for larger *ω* due to the “forgetfulness” of the network caused by the leak term in the ODE. At low frequencies, the recurrent drive ∝ *K* ⊛ *J* ***δm***(*t*) therefore partly cancels the direct drive ∝ *h*_ext_ sin (*ωt*). This is because the rate response *δm* is in phase, and the feedback *KJ* < 0 in the network is negative. The cancellation becomes less efficient at larger frequencies, because the recurrent drive asymptotically decays like *ω*^−1^ and is phase-shifted; the mean activity is low-pass-filtered Eq (9). The direct drive, in contrast, does not depend on the driving frequency *ω*. Therefore, the *S*_*m*_-term is low-pass-filtered twice and the *S*_*h*_-term term only once, therefore their sum has a peak at an intermediate frequency, as visible in Fig 3C, purple curve. Note that this cancellation generally appears in the balanced state, because the network feedback is always effectively inhibitory. Furthermore, the modulated-autocovariances-drive only vanishes for ${\bar{m}}_{\alpha }=\frac{1}{2}$; for realistic activity ${\bar{m}}_{\alpha }\ll \frac{1}{2}$ it is in anti-phase with *δm*(*t*), because it is defined including *W* < 0, which flips the phase by *π*.

Average covariances in inhibitory networks are negative [13]. As a consequence, in the setting of a single inhibitory population there is a second kind of cancellation: The two terms $\bar{c}$ and $N^{-1}\bar{a}$ in the prefactor $\bar{c}+N^{-1}\bar{a}$ of the susceptibility terms in Eq (12) partly cancel; their sum in fact vanishes in the large *N* limit [cf. 13, eq. (32) and their Fig 5]. This leads to the dominance of the *a*-term, shown in Fig 3D (orange curve). The maximum in the *S*-terms is therefore overshadowed by the *a*-term, which asymptotically also shows a second order low pass characteristics with ∝ *ω*^−2^. So in the purely inhibitory network the peak is not visible in the sum of all contributions to $\hat{C}\left(\omega \right)$.

In summary, the model of a single population in the balanced state exposes several generic features of time-dependent mean activities and covariances: Mean activities and the direct drive contribution to covariances follow the external modulation with first order low pass characteristics. The *S*_*m*_-term and the *a*-term of the covariances, being mediated by the mean activity, consequently expose a second order low pass filtering. The direct drive and the recurrent drive (the susceptibility terms) to large extent cancel at low frequencies, but not at high ones. Due to their overall decay in amplitude with increasing frequency, an intermediate maximum arises in their sum. In the single population model this peak is typically overshadowed by the *a*-term. This is because of the suppression of population fluctuations by negative feedback in the stationary state [10], which causes a small population variance $N^{-1}\bar{a}+\bar{c}$ and the latter term controls the amplitude of the susceptibility terms.

#### Two homogeneously connected populations

A slightly more realistic, but still simple setup is an EI-network with the same input for the inhibitory and the excitatory neurons, as studied before, in [13, parameters, except *m_X_* as in fig. 6 there]. This network is also inhibition-dominated, therefore we observe qualitatively the same competition of the two *S*-terms leading to the existence of a maximum in the *ω*-dependence of |*C*_1_|. In contrast to the single population case, in the E-I network the peak may be visible. This is because—in contrast to the single population case—covariances in this setup may also be positive, preventing the cancellation with the variances in the term $\bar{c}+\frac{\bar{a}}{N}$ that drives the *S*-terms. The latter contribution may therefore dominate over the *a*-term at small *ω*. Its dominance increases the larger the covariances are, which for example arises when increasing the external drive or by lowering the noise level at the input to the neurons. The “resonance” effect itself increases for weaker the excitatory synapses.

**Fig 4:**
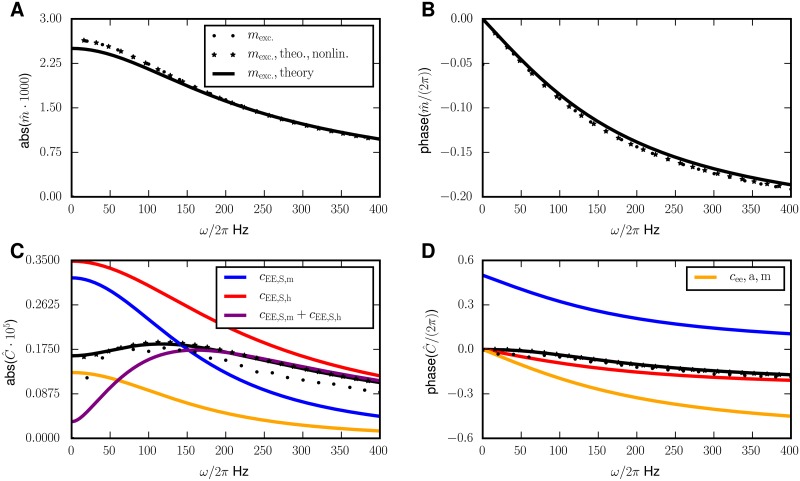
Periodically driven E-I network. **A** Amplitude of modulation of the mean activity deviating from the stationary value for the excitatory population. **B** Phase of the modulation of the mean activity. **C** Different contributions to the amplitude of the covariance between pairs of excitatory cells in dependence of the frequency *ω* of the external drive. **D** Phase of covariances relative to the driving signal. Analytical theory (Eqs (9) and (39)) shown by solid black curves, numerical solutions of the full mean field equations (Eqs (5) and (6)) (stars) and simulation results (dots, only indicated in the legend of A). Same color code as in **C**. In **C** and **D**, the contributions to the variation of covariances are shown separately: The *S*_*h*_-term in red, the *S*_*m*_-term in blue, their sum in purple and the *a*-term in yellow. The legend for **C** and **D** is split over both panels. Numerical solutions obtained by the same methods as in Fig 3. Parameters: *N*_*E*_ = *N*_*I*_ = *N*_*X*_ = 8192, *p*_*E*_ = *p*_*I*_ = *p*_*X*_ = *p* = 0.2, *m*_*E*_ = *m*_*I*_ ≈ 0.11, *m*_*X*_ = 0.25, identical to [13, e.g. fig. 6].

Fig 4C, indeed shows a peak of the response of the covariances at a frequency of about 120 Hz. We here focus on the covariances between excitatory neurons, because their activities are recorded most often and also cell assemblies are normally assumed to consist of excitatory neurons.

#### Two populations with inhomogeneous connections

**Fig 5:**
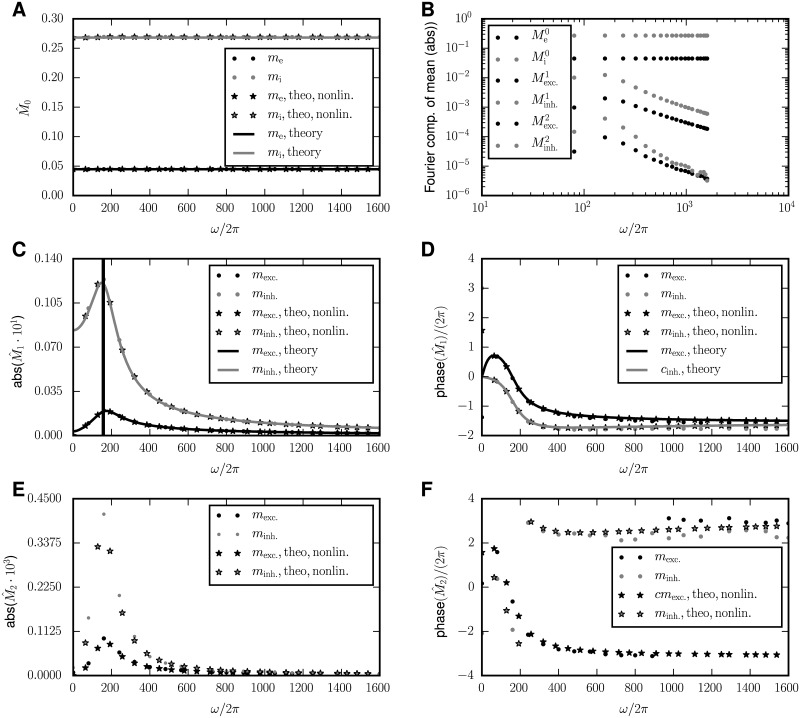
Driven E-I network with biologically inspired parameters: Mean activity. From the first to the third row, the zeroth to second Fourier mode of the mean activity is shown. **A** Constant part of mean activity (zeroth order). **B** First three Fourier-modes of the mean activities on a loglog-scale. **C** Amplitude of first mode of the mean activity. **D** Phase of first mode relative to driving signal. **E** and **F** are structured analogous to **C** and **D** for the second Fourier modes. Solid curves indicate the linear theory (Eq (9)), stars numerical integration of the full mean field equations (Eqs (5) and (6)) and dots the simulation results of the full network. Black symbols indicate the activity of excitatory, gray symbols of inhibitory neurons. Numerical results obtained by the same methods as in Fig 3. Noise amplitudes *σ*_noise,*E*_ = *σ*_noise,*I*_ = 10, *σ*_network,*E*_ = 2.8, *σ*_network,*E*_ = 4.6, other parameters of the network model given in Table 1.

The example of homogeneous connectivity helps to explain the fundamental mechanisms that shape the covariances; it is, however, certainly not very realistic. Furthermore, in the case of synaptic weights being different for individual receiving populations, the linearized connectivity *W* can have a pair of complex eigenvalues, which is qualitatively different to the setup described before. To check if the theory also works for parameters satisfying biological constraints, we choose the connectivity and activity levels in accordance to experimental studies. Apart from the results from [26], the parameters were measured in the layer 2/3 in the barrel cortex of mice. We select this layer, because it is the assumed location of cell assemblies [42], allowing us to relate our results to the original hypothesis of excess synchrony by activation of assemblies [26], a feature that could be considered in future studies. The connection probabilities are taken from [43], the fractions of excitatory and inhibitory neurons from [44] and the membrane time constant is extracted from [45, supplementary material]. We adjust the neurons’ thresholds such that the stationarity condition $\varphi \left(\bar{m}\right)=\bar{m}$ is fulfilled for *m*_*α*_ = *τν*_*α*_, where *α* ∈ {exc., inh.}, *ν*_*α*_ is the firing rate of the respective population and *τ* is the neuronal time constant. Note that the mapping *m* = *τν* implies a slightly different notion of a “spike” of a binary neuron than previously used [28]. The two conventions agree in the limit of vanishing firing rates (cf. section II B in S1 Text). The firing rate of 18 Hz given in [26] presumably reflects the activity of excitatory neurons (private communication). To obtain the firing rate of the inhibitory neurons *ν*_inh_., we scale the measurement from [26] by the ratio *ν*_inh_/*ν*_exc_. from [44]. All parameters are summarized in Table 1. The effective connectivity *W* of this system has two conjugate complex eigenvalues. Therefore, there exists a resonance frequency also for the mean activity, shown in Fig 5C.

**Table 1:** Parameters for the biologically inspired network model used in Figs 5 and 6 and Figs A, B, C and D in S1 Text.

		exc.	inh.	*ν* (Hz)	mean act.	#neurons	
exc.	connection prob.	0.168	0.5	18	0.045	1691	*τ* = 2.5 ms
	synaptic weight	0.37	−0.52				
inh.	connection prob.	0.327	0.36	108	0.27	230	*m*_ext_ = 0.1
	synaptic weight	0.82	−0.54				

**Fig 6:**
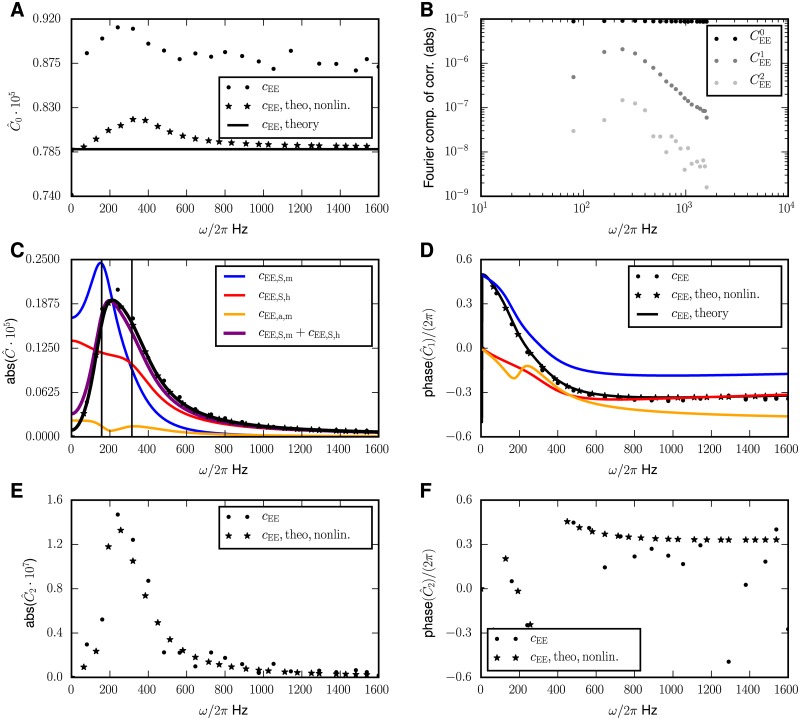
Driven E-I network with biologically inspired parameters: EE-Covariance. Response of the covariance to a perturbation with frequency *ω* in the Fourier space. **A** Zeroth Fourier mode (time independent part) of the covariance. **B** Absolut value of the first three Fourier components of the *c*_*ee*_-covariances on a loglog-scale. **C** Absolute value of the first order of the time-dependent part of the covariance. **D** Phase angle in relation to the driving signal. **E** and **F** are analogous to **C** and **D** for the second Fourier modes. Solid lines indicate the linear theory Eq (39), stars the results of the numerical solution of the full mean-field theory Eqs (5) and (6) and dots the direct simulation of the full network. Numerical results obtained by the same methods as in Fig 3. Parameters of the network model as in Fig 5.

In the two upper panels of Figs 5 and 6, we compare the stationary values for the mean activity Eq (7) and the covariances Eq (8) with the respective time averaged results of the simulation and with the numerical solution of the full mean-field equations. The stationary statistics have been investigated before for other parameters in finite networks [13] and in the limit *N* → ∞ [6]. The second harmonics extracted from the simulations and the numerical solution of the full mean-field equations show good agreement and are overall small compared to the zeroth and first harmonics, justifying the truncation of the Fourier series in the analytical theory after the first term.

The first harmonics of the mean activity (see Fig 5) and covariances (see Fig 6) predicted by the linear response theory agree well with simulations and the numerical solution. This is not necessarily clear a priori because the perturbation in the input to every neuron is of the order $O\left(\frac{\sigma }{10}\right)$, where *σ* is the input noise level of the unperturbed system. However, linear response theory works surprisingly well, even for the covariances caused by a perturbation leading to a response of the same order of magnitude as the stationary value. Increasing the perturbation strength *h*_ext_ further ultimately leads to a breakdown of the linear perturbation theory, visible in the growing absolute values of the second Fourier modes of mean activities and covariances (Fig D in S1 Text). The maximal modulation in the firing rates amounts to ≈ 0.8 Hz for the excitatory and 4.9 Hz for the inhibitory neurons.

In this biologically inspired setting, it is also interesting to apply the Unitary Event (UE) analysis to our data, as it was done for experimental data in [26]. Because this is a bit aside the scope of this paper, we present this part in the appendix, Sec. I in S1 Text.

The connectivity matrix has complex eigenvalues $\lambda_{1}=\lambda_{2}^{*}$, so we observe a resonance of the mean activities at the frequencies

$$\begin{array}{c} f_{\text{res},\text{mean}}=\frac{ℑ\left(\lambda_{1}\right)}{\tau 2\pi }, \end{array}$$

indicated by a vertical line in Fig 5C. The components of *δm* are composed of different modes, therefore their maximum does not appear exactly at *f*_res, mean_. The covariances are shaped by more modes: In general, the covariance matrix for a three-dimensional quantity has 6 independent components. In our case, *c*_*XX*_ is always 0, which is a consequence of the missing feedback to *X*. Now, the evolution of every mode of $\tilde{\delta c}$ is given by the sum of two eigenvalues of 1 − *W*, i.e. 2 − λ, 2 − λ*, 2 − 2λ, 2 − 2λ* and 2 − (λ* + λ). The missing mode is the “trivial” one owing to the vanishing eigenvalue of *W*. So the behavior of the “kernel” of the ODE for *δc* is given by the resonances at $\frac{\left|ℑ\left(\lambda \right)\right|}{\tau 2\pi }$ and $2\cdot \frac{\left|ℑ\left(\lambda \right)\right|}{\tau 2\pi }$. In addition, the inhomogeneity of the ODE (10) (its right hand side) is already resonant at $\frac{\left|ℑ\left(\lambda \right)\right|}{\tau 2\pi }$. All these modes are mixed with different strength in the different modes of *δc*, giving rise to a maximum of |*C*_1_| somewhere in the vicinity of *f*_res, mean_ and 2*f*_res, mean_. In all cases the “resonances” are damped, therefore, a resonance catastrophe, induced by *δm* oscillating with the resonance frequency of *δc*, cannot occur. We also notice that all resonances are the stronger, the closer ℜ(λ) is to 1, the critical point, which makes sense intuitively: The damping comes from the overall inhibitory feedback; at the critical point the leak term is exactly compensated by positive feedback of identical magnitude. It is worth noticing that the effect of the partial cancellation of the *S*-terms, which can be read off from Eq (10) and is described in the previous subsections for small *ω* is still valid. The functional form of |*C*_1_(*ω*)|, however, is now mainly determined by the resonances due to the complex eigenvalues of *W*.

The *ω*-dependencies of the *c*_II_- and the *c*_EI_- covariances shown in the appendix are qualitatively similar (Fig. B and Fig. C in S1 Text). The stationary covariance is well predicted by the theory [13], which is confirmed here.

**Fig 7:**
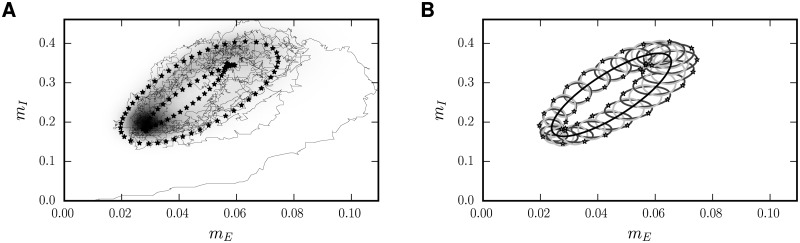
Distribution of population-averaged activity of periodically driven E-I network. **A** Empirical density of population activity of the E-I network. Gray shading indicates time-averaged occupation of states. The thin mid gray curve is a sample of the binary dynamics of 10 periods after the start of the simulation. The black dots indicate the *σ*-region predicted by the linear theory as described by Eq (13) in the main text. **B** Limit cycle of the linear theory (black ellipse), together with error ellipses stemming from the sum of covariances and variances (dark gray, slightly tilted) and representing solely variances (light gray). The stars are at the same places as in A. Parameters are given in Table 1, only the perturbation strength is increased to *h*_ext_ = 6 (noise level around *σ*_*E*_ ≃ 14, *σ*_*I*_ ≃ 23) for reasons of readability (for this value the simulated results already show deviations from the linear approximation as shown in Fig. D in S1 Text). The perturbing frequency is chosen to be *f* = 80 Hz.

Fig 7 illustratively summarizes the results of this section. In panel A, the probability of the binary system to be in a certain activity state (*m*_inh_, *m*_exc_)^T^ is indicated by different gray shades, the darker, the higher the probability to find it in the respective area. On top, the area including the most probable network states, as predicted by the linear theory, is indicated by black dots. Its construction is depicted in panel B: We draw the limit cycle (black) formed by the points (〈*m*_inh_(*t*)〉, 〈*m*_exc_(*t*)〉)^T^ as a parametric plot with time as parameter. Then, we define the points on the error ellipse (*m*_inh_, *m*_exc_)^T^ as follows

$$\begin{array}{c} \delta m{\left(t\right)}^{T}{\left(c^{\text{pop}}\left(t\right)\right)}^{-1}\delta m\left(t\right)=1, \end{array}$$ (13)

where *δ**m***^*T*^ := (*m*_inh_, *m*_exc_, 0)^T^ − (〈*m*_inh_〉, 〈*m*_exc_〉, 0)^T^ and

$$\begin{array}{c} c^{\text{pop}}\left(t\right)=\left(\begin{array}{ccc} c_{\text{EE}}^{\text{pop}}\left(t\right) & c_{\text{EI}}^{\text{pop}}\left(t\right) & c_{\text{EX}}^{\text{pop}}\left(t\right)\\ c_{\text{EI}}^{\text{pop}}\left(t\right) & c_{\text{II}}^{\text{pop}}\left(t\right) & c_{\text{IX}}^{\text{pop}}\left(t\right)\\ c_{\text{EX}}^{\text{pop}}\left(t\right) & c_{\text{IX}}^{\text{pop}}\left(t\right) & 0 \end{array}\right). \end{array}$$

In this way, the solutions *δ**m***(*t*) of Eq (13) are composed of all points that are one standard deviation away from the expected activity. The covariances enter the total population averaged variability, given by

$$\begin{array}{ccc} c_{\alpha \beta }^{\text{pop}}\left(t\right) & ≔ & \left\langle \delta m_{\alpha }\;\left(t\right)\delta m_{\beta }\;\left(t\right)\right\rangle =\left\langle \frac{1}{N_{\alpha }}\sum_{i\in \alpha }\delta n_{i}\;\left(t\right)\frac{1}{N_{\beta }}\sum_{i\in \beta }\delta n_{i}\;\left(t\right)\right\rangle \\ & = & \frac{\delta_{\alpha \beta }}{N_{\alpha }^{2}}\sum_{i\in \alpha }\left\langle \delta n_{i}^{2}\left(t\right)\right\rangle +\frac{1}{N_{\alpha }N_{\beta }}\sum_{i\in \alpha ,\;j\in \beta ,i\neq j}\left\langle \delta n_{i}\;\left(t\right)\delta n_{j}\;\left(t\right)\right\rangle \\ & \approx & \delta_{\alpha \beta }\frac{a_{\alpha }\;\left(t\right)}{N_{\alpha }}+c_{\alpha \beta }\;\left(t\right) \end{array}$$

with the definitions Eqs (3) and (4).

The two points on the border of the dark gray error-ellipses of the full covariances with the largest distance to the tangent of the limit cycle at (〈*m*_inh_〉, 〈*m*_exc_〉) are marked by a star, which, taken together, form the border of a tube-shaped *σ*-area. This tube indicates the region in which we most likely expect to find the system. To visualize the contributions of auto- and pairwise covariances, we plot in light gray the error ellipses based solely on the variances (*c*^pop^ (*t*) is diagonal in this case). The dark error ellipses are bigger than the light ones, indicating that the covariances are positive and their axes are tilted; the *c*_*EI*_ = *c*_*IE*_-component is nonzero. Furthermore, the error ellipses significantly change their size in time, indicative of the modulation of the fluctuations with time. The variances grow monotically with the respective mean activities, explaining that the light gray ellipses are largest (smallest) where the mean activities are largest (smallest). One can read off the phase shift of *c*_*EE*_ to *m*_*E*_ to be roughly $\frac{\pi }{2}$: the deviation of the dark gray error ellipses from the light gray ones is largest at the points where $m_{E}\;\left(t\right)\approx {\bar{m}}_{E}$ and *δm*_*I*_ (*t*) is minimal.

## Methods

### Glauber dynamics in mean-field theory

We have left out so far several steps in the derivation of the results that were not necessary for the presentation of the main ideas. In this section, we will therefore give a self-contained derivation of our results also necessitating paraphrases of some results known from earlier works. The starting point is the master equation for the probability density of the possible network states emerging from the Glauber dynamics [34] described in “*Binary network model and its mean field equations*” (see for the following also [13, 37])

$$\begin{array}{c} \frac{\partial p}{\partial t}\left(n,t\right)=\underset{\text{update}\;\text{rate}}{\underset{︸}{\frac{1}{\tau }}}\sum_{i=1}^{N}\underset{\in \{-1,1\},\text{direction}\;\text{of}\;\text{flux}}{\underset{︸}{\left(2n_{i}-1\right)}}\;\underset{\text{net}\;\text{flux}\;\text{due}\;\text{to}\;\text{neuron}\;i}{\underset{︸}{\phi_{i}\left(n\setminus n_{i},t\right)}}\;\forall \;n\in {\left\{0,1\right\}}^{N}, \end{array}$$ (14)

where

$$\begin{array}{ccc} \phi_{i}\left(n\setminus n_{i},t\right) & = & \underset{\text{neuron}\;i\;\text{transition}\;\text{up}}{\underset{︸}{p\left(n_{i-},t\right)\;F_{i}\left(n_{i-}\right)}}-\underset{\text{neuron}\;i\;\text{transition}\;\text{down}}{\underset{︸}{p\left(n_{i+},t\right)\;\left(1-F_{i}\left(n_{i+}\right)\right)}}\\ & = & -p\left(n_{i+}\right)+p\left(n_{i-},t\right)\;F_{i}\left(n_{i-}\right)+p\left(n_{i+},t\right)\;F_{i}\left(n_{i+}\right). \end{array}$$

The activation function *F*_*i*_(***n***) is given by Eq (1).

Using the master equation (for details cf. section II A in S1 Text), one can derive a differential equation for the mean activity of the neuron *i*, 〈*n*_*i*_〉 (*t*) = ∑_***n***_ *p*(***n***, *t*)*n*_*i*_ and the raw covariance of the neurons *i* and *j*, 〈*n*_*i*_ (*t*) *n*_*j*_ (*t*)〉 = ∑_***n***_ *p*(**n**, *t*)*n*_*i*_*n*_*j*_ [6, 13, 27, 34, 37]. This yields

$$\begin{array}{ccc} \tau \frac{d}{dt}\left\langle n_{k}\right\rangle \;\left(t\right) & = & -\left\langle n_{k}\right\rangle \;\left(t\right)+\left\langle F_{k}\;\left(t\right)\right\rangle \\ \frac{d}{dt}\left\langle n_{k}\;\left(t\right)n_{l}\;\left(t\right)\right\rangle & = & \left\{-\left\langle n_{k}\;\left(t\right)n_{l}\;\left(t\right)\right\rangle +\left\langle n_{l}\;\left(t\right)\;F_{k}\;\left(t\right)\right\rangle \right\}+\left\{k↔l\right\}. \end{array}$$ (15)

As mentioned in “*Binary network model and its mean field equations*”, we assume that the input *h*_*i*_ coming from the local and the external population is normally distributed, say with mean *μ*_*i*_ and standard deviation *σ*_*i*_ given by

$$\begin{array}{cc} \mu_{i}\left(t\right) & ≔\left\langle h_{i}\right\rangle ={\left(J\;\left\langle n\right\rangle \right)}_{i}+h_{\text{ext}}\text{ sin}\left(\omega t\right)\\ \sigma_{i}^{2}\left(t\right) & ≔\left\langle h_{i}^{2}\right\rangle -{\left\langle h_{i}\right\rangle }^{2}=\sum_{k,k^{\prime }=1}^{N}J_{i,k}J_{i,k^{\prime }}\left(\left\langle n_{k}n_{k^{\prime }}\right\rangle -\left\langle n_{k}\right\rangle \left\langle n_{k^{\prime }}\right\rangle \right)+{\left(\sigma_{i}^{\text{noise}}\right)}^{2}\\ & ={\left(J^{T}\;cJ\right)}_{ii}+J\;⊛\;J\left\langle n\right\rangle \;⊛\;\left(1-\left\langle n\right\rangle \right)+{\left(\sigma_{i}^{\text{noise}}\right)}^{2}, \end{array}$$ (16)

where the average 〈〉 is taken over realizations of the stochastic dynamics and we used the element-wise (Hadamard) product (see main text).

**Fig 8:**
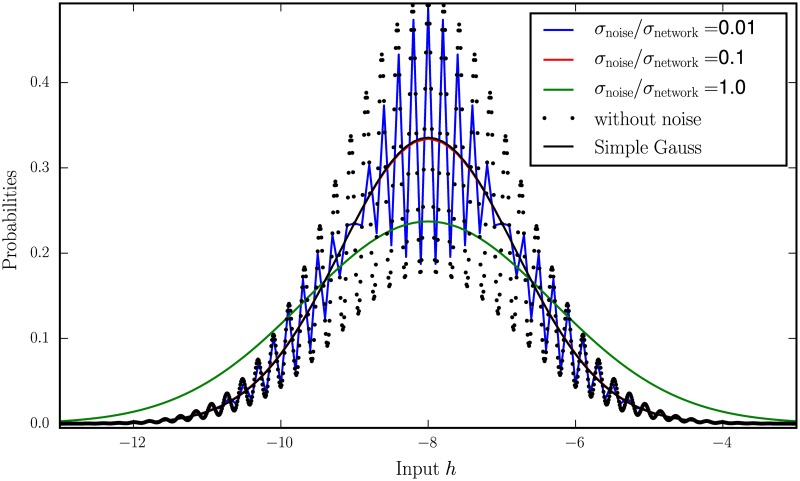
Distribution of inputs from binary neurons for different noise levels. Probability distribution of synaptic input *h*_*i*_ = ∑_*j*_ *J*_*ij*_*n*_*j*_ + *ξ*_*i*_ of a neuron in a network of independently active cells *n*_*j*_ with 〈*n*_*E*_〉 = 〈*n*_*I*_〉 = 0.2 and synaptic weights *j*_*I*_ = −0.21, *j*_*E*_ = 0.01. $\left|\frac{j_{E}}{j_{I}}\right|$ was deliberately chosen to be large because only then the convolution of a binomial distribution “squeezed” to the step size *j*_*E*_ with the binomial distribution squeezed to the step size |*j*_*I*_| results in a probability distribution with many local maxima leading to the impression of an oscillation. The noiseless case *ξ*_*i*_ = 0 is shown as black dots. The solid black curve indicates the Gaussian approximation (cf. e.g. Eq (16), here without perturbation) of this distribution from the main text. This distribution appears in the expectation values of the activation function *F* (cf. e.g. Eq (1)): It is a Gaussian distribution with the mean *μ* = *K*_*E*_ *j*_*E*_ *m*_*E*_ + *K*_*I*_*j*_*I*_ *m*_*I*_ and the variance $\sigma_{\text{network}}^{2}=K_{E}j_{E}^{2}m_{E}\;\left(1-m_{E}\right)+K_{I}j_{I}^{2}m_{I}\;\left(1-m_{I}\right)$ of the original binomial distributions Binom(m_E_, K_E_), Binom(m_I_, K_I_). The other curves indicate convolutions with the Gaussian noise $\xi ∼N(0,\sigma_{noise})$ of different magnitudes *σ*_noise_, given in units of the noise level *σ*_network_ intrinsically produced by the network.

The additional noise introduced in Eq (1) effectively leads to a smoothing of the neurons’ activation threshold and broadens the width of the input distribution. It can be interpreted as additional variability coming from other brain areas. Furthermore, it is computationally convenient, because the theory assumes the input to be a (continuous) Gaussian distribution, while in the simulation, the input $\sum_{l=k}^{N}J_{ik}n_{k}$, being a sum of discrete binary variables, can only assume discrete values. The smoothing by the additive noise therefore improves the agreement of the continuous theory with the discrete simulation. Already weak external noise compared to the intrinsic noise is sufficient to obtain a quite smooth probability distribution of the input (Fig 8).

The description in terms of a coupled set of moment equations instead of the ODE for the full probability distribution here serves to reduce the dimensionality: It is sufficient to describe the time evolution of the moments on the population level, rather than on the level of individual units. To this end we need to assume that the synaptic weights *J*_*ij*_ only depend on the population *α*, *β* ∈ {exc., inh., ext.} that *i* and *j* belong to, respectively, and thus (re)name them *J*_*αβ*_ (homogeneity). Furthermore, we assume that not all neurons are connected to each other, but that *K*_*αβ*_ is the number of incoming connections a neuron in population *α* receives from a neuron in population *β* (fixed in-degree). The incoming connections to each neuron are chosen randomly, uniformly distributed over all possible sending neurons. This leads to expressions for the population averaged input *h*_*α*_, mean activity *m*_*α*_ and covariance *c*_*αβ*_, formally nearly identical to those on the single cell level and analogous to those in [13, sec. Mean-field solution].

#### Mean activity: Stationary part and response to perturbation in linear order

We are now able to calculate the quantity 〈*F*_*α*_ (***n***(*t*), *t*)〉 = 〈*H* (*h*_*α*_ (*t*) − *θ*)〉 (recall that *h*_*α*_ (*t*) is a Gaussian random variable with mean *μ*_*α*_ (*t*) and standard deviation *σ*_*α*_ (*t*)), the nonlinearity of the ODEs 15 on the population level. Multiplying *H* (*h*_*α*_ (*t*) − *θ*_*α*_) by the Gaussian probability density for *h*_*α*_ (*t*), we get, after substitution of the integration variable,

$$\begin{array}{cc} & \left\langle F_{\alpha }\;\left(n\;\left(t\right),t\right)\right\rangle =\left\langle H\;\left(h_{\alpha }\;\left(t\right)-\theta_{\alpha }\right)\right\rangle \\ = & \frac{1}{\sqrt{\pi }}\int_{\frac{\theta -\mu_{\alpha }\left(t\right)}{\sqrt{2}\sigma_{\alpha }\left(t\right)}}^{\infty }e^{-x^{2}}\;dx=\frac{1}{2}\text{erfc }\left(\frac{\theta_{\alpha }-\mu_{\alpha }\;\left(t\right)}{\sqrt{2}\sigma_{\alpha }\;\left(t\right)}\right)\\ ≕ & \varphi \left(\mu_{\alpha }\left(m\;\left(t\right),h_{\text{ext}}\text{ sin }\left(\omega t\right)\right)\right),\sigma {}_{\alpha }\left(m\;\left(t\right),c\;\left(t\right)\right)), \end{array}$$ (17)

where we defined the average input *μ*_*α*_ and the width of the input distribution *σ*_*α*_

$$\begin{array}{ccc} \mu_{\alpha }\left(t\right) & ≔ & {\left[\left(K\;⊛\;J\right)\;m\;\left(t\right)\right]}_{\alpha }+h_{\text{ext}}\text{sin}\left(\omega t\right) \end{array}$$ (18)

$$\begin{array}{ccc} \sigma_{\alpha }^{2}\left(t\right) & ≔ & {\left[{\left(K\;⊛\;J\right)}^{T}\;c\left(t\right)\;\left(K\;⊛\;J\right)\right]}_{\alpha \alpha }\\ & & +{\left[K\;⊛\;J\;⊛\;J\;m\;\left(t\right)\;⊛\;\left(1-m\;\left(t\right)\right)\right]}_{\alpha }+\sigma_{\alpha ,\text{noise}}^{2}. \end{array}$$ (19)

Recall that we defined $\bar{x}$ to be the quantity *x* in the stationary case (without external input). For the linear approximation around $\mu_{\alpha }={\bar{\mu }}_{\alpha }$, $\sigma_{\alpha }={\bar{\sigma }}_{\alpha }$ and *h*_ext_ = 0, we have to take into account all dependencies via inner derivatives. We set $\delta \mu_{\alpha }=\mu_{\alpha }-{\bar{\mu }}_{\alpha }$ and $\delta \sigma_{\alpha }=\sigma_{\alpha }-{\bar{\sigma }}_{\alpha }$. Note that *δμ* includes the variation of *μ* both because of fluctuations in the network and because of the external drive. The Taylor expansion up to linear order is

$$\begin{array}{c} \varphi \left(\mu {}_{\alpha }\left(m,c,h_{\text{ext}}\right),\sigma_{\alpha }\left(m,c\right)\right)\approx \varphi \left({\bar{\mu }}_{\alpha },{\bar{\sigma }}_{\alpha }\right)+\underset{≕{\bar{S}}_{\alpha }}{\underset{︸}{S\left({\bar{\mu }}_{\alpha },{\bar{\sigma }}_{\alpha }\right)}}\left[\delta \mu_{\alpha }+\frac{\theta -{\bar{\mu }}_{\alpha }}{{\bar{\sigma }}_{\alpha }}\delta \sigma_{\alpha }\right], \end{array}$$

where we introduced the susceptibility on the population level

$$\begin{array}{c} S\;\left(\mu_{\alpha }\;\left(t\right),\sigma_{\alpha }\;\left(t\right)\right)\;≔\;\frac{d}{d\mu_{\alpha }\;\left(t\right)}\varphi \left(\mu_{\alpha }\left(t\right),\sigma {}_{\alpha }\left(t\right)\right)=\frac{1}{\sqrt{2\pi }\sigma_{\alpha }\left(t\right)}\;e^{-\frac{{\left(\mu_{\alpha }\left(t\right)-\theta_{\alpha }\right)}^{2}}{2\sigma_{\alpha }^{2}\left(t\right)}}. \end{array}$$ (20)

Now, we express *δσ*_*α*_ and *δμ*_*α*_ via $\delta m\;≔\;m-\bar{m}$ and $\delta c\;≔\;c-\bar{c}$ (cf. [13, eq. (29)] for the time-independent case):

$$\begin{array}{ccc} \delta \mu_{\alpha }\;\left(t\right) & = & \sum_{\beta }K_{\alpha \beta }J_{\alpha \beta }\delta m_{\beta }\;\left(t\right)+h_{\text{ext}}\text{ sin }\left(\omega t\right) \end{array}$$ (21)

$$\begin{array}{ccc} \delta \sigma_{\alpha }\;\left(t\right) & = & \frac{1}{2\sigma_{\alpha }}\left(\sum_{\beta }K_{\alpha \beta }J_{\alpha \beta }^{2}\left(1-2m_{\beta }\right)\delta m_{\beta }\;\left(t\right)+\sum_{\beta ,\gamma }K_{\alpha \beta }K_{\alpha \gamma }J_{\alpha \beta }J_{\alpha \gamma }\delta c_{\beta \gamma }\;\left(t\right)\right). \end{array}$$ (22)

Note that in *δμ*_*α*_ (but not *δσ*_*α*_), the perturbation occurs again explicitly. In Eq (33), we demonstrate that *δc* scales like $\frac{\delta m}{N}$, like in the stationary case. Furthermore, we certainly have $\frac{\left|K\right|}{N}=O\left(1\right)$ and $\sigma =O\left(\sqrt{\left|K\right|}\right)$, thus

$$\begin{array}{c} \left|\delta \mu \;\left(t\right)\right|=O\left(\left|K\right|\left|\delta m\;\left(t\right)\right|\right)=O\left(h_{\text{ext}}\right),\;\text{but}\;\left|\delta \sigma \;\left(t\right)\right|=O\left(\sqrt{\left|K\right|}\left|\delta m\;\left(t\right)\right|\right)=O\left(\frac{h_{\text{ext}}}{\sqrt{\left|K\right|}}\right). \end{array}$$

We therefore neglect *δ****σ*** in our calculations for *δ**m*** from Eq (24) on. This yields for the linearization of the ODE Eq (5)

$$\begin{array}{ccc} \tau \frac{\partial }{\partial t}\delta m_{\alpha }\;\left(t\right)+\delta m_{\alpha }\;\left(t\right) & = & {\bar{S}}_{\alpha }\;\left[\delta \mu_{\alpha }\;\left(t\right)+\right.\underset{={\text{erfc}}^{-1}\left(\bar{m}\right)}{\underset{︸}{\frac{\theta_{\alpha }-{\bar{\mu }}_{\alpha }}{{\bar{\sigma }}_{\alpha }}}}\left.\delta \sigma_{\alpha }\;\left(t\right)\right] \end{array}$$ (23)

$$\begin{array}{ccc} \tau \frac{\partial }{\partial t}\delta m_{\alpha }\;\left(t\right)+\delta m_{\alpha }\;\left(t\right) & = & \sum_{\beta }W_{\alpha \beta }\delta m_{\beta }\;\left(t\right)+{\bar{S}}_{\alpha }h_{\text{ext}}\text{ sin }\left(\omega t\right)+O\left(h_{\text{ext}}^{2},\frac{1}{\sqrt{K}}\right), \end{array}$$ (24)

where we used the relation $\frac{\Theta_{\alpha }-{\bar{\mu }}_{\alpha }}{{\bar{\sigma }}_{\alpha }}=\sqrt{2}{erfc}^{-1}\;\left(2{\bar{m}}_{\alpha }\right)$, derived from Eq (7) in connection with Eq (17), which implies that this expression does not depend on *K*, but solely on ${\bar{m}}_{\alpha }$ and we defined

$$\begin{array}{c} W_{\alpha \beta }\;≔\;{\bar{S}}_{\alpha }K_{\alpha \beta }J_{\alpha \beta }. \end{array}$$

The only change compared to the setup in [13] is again the occurrence of a periodic term, here ${\bar{S}}_{\alpha }h_{\text{ext}}\text{ sin}(\omega t)$ sin (*ωt*).

We solve Eq (24) by transforming it into the eigenbasis of the matrix *W*_*αβ*_

$$\begin{array}{c} U^{-1}WU=\text{diag }\left(\lambda_{1},..,\lambda_{\tilde{N}}\right)\;≔\;\Lambda . \end{array}$$ (25)

We multiply Eq (24) by *U*^−1^, define *δm*^*α*^ ≔ (*U*^−1^)^*αβ*^ *δm*_*β*_ and get

$$\begin{array}{c} \tau \frac{d}{dt}\delta m^{\alpha }=-\delta m^{\alpha }+\Lambda_{\beta }^{\alpha }\delta m^{\beta }+{\left(U^{-1}\right)}^{\alpha \beta }{\bar{S}}_{\beta }h_{\text{ext}}\text{sin }\left(\omega t\right). \end{array}$$ (26)

Note that the input is projected onto the respective eigenmodes. Eq (26) can be solved including the transient phase by the method of variation of constants.

But as we are only interested in the cyclostationary part of the solution, we can neglect the solution of the homogeneous part and solely compute the particular solution. Observe that $\frac{d}{dt}\text{Im}(\delta m^{\alpha }(t))=\text{Im }\left(\frac{d}{dt}\delta m^{\alpha }(t)\right)$ for a differentiable function *δm*^*α*^ because t∈ℝ. We insert the ansatz $\delta m^{\alpha }=M_{1}^{\alpha }e^{i\omega t}$ and solve for $M_{1}^{\alpha }$, which gives Eq (9) of the main text. For further calculations, keep in mind that $M_{\alpha }^{1}$ and therefore *δm*_*α*_ are of order $O\left(h_{\text{ext}}\frac{S}{SKJ}\right)=O\left(h_{\text{ext}}\frac{1}{KJ}\right)$. In section II C in S1 Text, we describe how to extract the right phase of the real solution from the complex ansatz.

#### Covariances: Stationary part and response to a perturbation in linear order

Using Eq (15) in the population-averaged version, we calculate the derivative of the zero time-lag covariance

$$\begin{array}{c} c_{\alpha \beta }\;\left(t\right)\;≔\;\frac{1}{N_{\alpha }N_{\beta }}\sum_{i\in \alpha ,j\in \beta ,i\neq j}\left\langle n_{i}\left(t\right)n_{j}\left(t\right)\right\rangle -\left\langle n_{i}\left(t\right)\right\rangle \left\langle n_{j}\left(t\right)\right\rangle \end{array}$$

getting

$$\begin{array}{c} \tau \frac{dc_{\alpha \beta }\;\left(t\right)}{dt}=-2c_{\alpha \beta }\;\left(t\right)+\frac{1}{N_{\alpha }N_{\beta }}\sum_{i\in \alpha ,j\in \beta ,i\neq j}\;\left\langle F_{j}\;\left(n\;\left(t\right)\right)\delta n_{i}\;\left(t\right)\right\rangle +\left\langle F_{i}\;\left(n\;\left(t\right)\right)\delta n_{j}\;\left(t\right)\right\rangle . \end{array}$$

Neglecting cumulants of order higher than two, we can expand the expectation value 〈*F*_*i*_ (***n*** (*t*)) *δn*_*j*_ (*t*)〉 (cf. [13, 33, section “Linearized equation for correlations and susceptibility”]) and get

$$\begin{array}{c} \left\langle F_{i}\;\left(n\;\left(t\right)\right)\delta n_{j}\;\left(t\right)\right\rangle \approx S\;\left(\mu_{i}\;\left(t\right),\sigma_{i}\;\left(t\right)\right)\;\sum_{k\neq j}J_{ik}c_{kj}\;\left(t\right)+S\;\left(\mu_{i}\;\left(t\right),\sigma_{i}\;\left(t\right)\right)\;J_{ij}a_{j}\;\left(t\right). \end{array}$$ (27)

After carrying out the population averaging, we get the ordinary differential equation

$$\begin{array}{cc} & \tau \frac{dc_{\alpha \beta }\;\left(t\right)}{dt}\\ = & \left\{-c_{\alpha \beta }\;\left(t\right)+\sum_{\gamma }S\;\left(\mu_{\alpha }\;\left(t\right),\sigma_{\alpha }\;\left(t\right)\right)\;K_{\alpha \gamma }J_{\alpha \gamma }\;\left(c_{\gamma \beta }\;\left(t\right)+\delta_{\gamma \beta }\frac{a_{\beta }\;\left(t\right)}{N_{\beta }}\right)\right\}+\;\left\{\alpha ↔\beta \right\}. \end{array}$$ (28)

Therefore, the stationary part $\bar{c}$ of the covariances fulfills the relation (cf. [13, 33])

$$\begin{array}{c} 2{\bar{c}}_{\alpha \beta }=\sum_{\gamma }S\;\left({\bar{\mu }}_{\alpha },{\bar{\sigma }}_{\alpha }\right)\;{\left(K\;\circ \;J\right)}_{\alpha \gamma }\;\left({\bar{c}}_{\gamma \beta }+\delta_{\gamma \beta }\frac{{\bar{a}}_{\beta }}{N_{\beta }}\right)+\alpha ↔\beta . \end{array}$$ (29)

As for the mean activities, we want to make a little step (of order *h*_ext_, to be precise) away from the stationary state determining the deviation $\delta c\;\left(t\right)\;≔\;c\;\left(t\right)-\bar{c}$. For that, we have to calculate the Taylor expansion of *S* (*μ*_*α*_ (*t*), *σ*_*α*_ (*t*)) in *δ**m***, i.e.

$$\begin{array}{cc} & S\;\left(\mu_{\alpha }\;\left(t\right),\sigma_{\alpha }\;\left(t\right)\right)\\ ≔ & \frac{1}{\sqrt{2\pi }}\frac{1}{\sigma_{\alpha }\;\left(t\right)}\text{exp }\left(-\;\frac{{\left(\mu_{\alpha }\;\left(t\right)-\theta_{\alpha }\right)}^{2}}{2\;{\left(\sigma_{\alpha }\;\left(t\right)\right)}^{2}}\right)\\ \approx & S\;\left({\bar{\mu }}_{\alpha },{\bar{\sigma }}_{\alpha }\right)+\left(\frac{\partial S}{\partial \mu_{\alpha }\;\left(t\right)}\delta \mu_{\alpha }+\frac{\partial S}{\partial \sigma_{\alpha }\;\left(t\right)}\delta \sigma_{\alpha }\right){|}_{\mu_{\alpha }={\bar{\mu }}_{\alpha },\sigma_{\alpha }={\bar{\sigma }}_{\alpha }}, \end{array}$$

where *δμ*_*α*_ and *δσ*_*α*_ are given by Eq (21) and

$$\begin{array}{ccc} \frac{\partial S}{\partial \mu_{\alpha }\left(t\right)}\left({\bar{\mu }}_{\alpha },{\bar{\sigma }}_{\alpha }\right) & = & \frac{\theta_{\alpha }-{\bar{\mu }}_{\alpha }}{{\bar{\sigma }}_{\alpha }^{2}}S\left({\bar{\mu }}_{\alpha },{\bar{\sigma }}_{\alpha }\right)\\ \frac{\partial S}{\partial \sigma_{\alpha }\left(t\right)}\left({\bar{\mu }}_{\alpha },{\bar{\sigma }}_{\alpha }\right) & = & -\frac{1}{{\bar{\sigma }}_{\alpha }}\left(1-{\left(\frac{\theta_{\alpha }-{\bar{\mu }}_{\alpha }}{{\bar{\sigma }}_{\alpha }}\right)}^{2}\right)S\left({\bar{\mu }}_{\alpha },{\bar{\sigma }}_{\alpha }\right)\\ & = & \frac{\theta_{\alpha }-{\bar{\mu }}_{\alpha }}{{\bar{\sigma }}_{\alpha }^{2}}\left(\underset{=O\left(1\right)}{\underset{︸}{\frac{\theta_{\alpha }-{\bar{\mu }}_{\alpha }}{{\bar{\sigma }}_{\alpha }}}}-\underset{=O\left(1\right)}{\underset{︸}{\frac{{\bar{\sigma }}_{\alpha }}{\theta_{\alpha }-{\bar{\mu }}_{\alpha }}}}\right)S\left({\bar{\mu }}_{\alpha },{\bar{\sigma }}_{\alpha }\right) \end{array}$$

Here again, the relation $\frac{\Theta_{\alpha }-{\bar{\mu }}_{\alpha }}{{\bar{\sigma }}_{\alpha }}=\sqrt{2}{erfc}^{-1}\;\left(2{\bar{m}}_{\alpha }\right)$ was used to estimate the dependence on *K*. We insert the linearization of *S* and the expressions for *δμ* and *δσ*, Eq (21), into the ODE for $c_{\alpha \beta }\;\left(t\right)={\bar{c}}_{\alpha \beta }+\delta c_{\alpha \beta }\;\left(t\right)$ to get, after neglecting the contributions of order $O\left(h_{\text{ext}}^{2}\right)$ and sorting the rest into terms proportional to *δc*, *h*_ext_ and *δm* respectively:

$$\begin{array}{cc} & \tau \frac{d}{dt}\delta c_{\alpha \beta }\;\left(t\right)+\left\{\sum_{\gamma }\;\left(\delta_{\alpha \gamma }-S\;\left({\bar{\mu }}_{\alpha },{\bar{\sigma }}_{\alpha }\right)K_{\alpha \gamma }J_{\alpha \gamma }\right)\delta c_{\gamma \beta }\;\left(t\right)\right\}+\left\{\alpha ↔\beta \right\}\\ = & \left\{\frac{\partial S}{\partial \mu_{\alpha }\;\left(t\right)}\sum_{\gamma }K_{\alpha \gamma }J_{\alpha \gamma }\;\left(\bar{c}{}_{\gamma \beta }+\frac{{\bar{a}}_{\beta }}{N_{\beta }}\delta_{\gamma \beta }\right)\right.\;\left(h_{\text{ext}}\text{ sin }\left(\omega t\right)+\underset{=O\;\left(h_{\text{ext}}\right)}{\underset{︸}{\sum_{\delta }K_{\alpha \delta }J_{\alpha \delta }\delta m_{\delta }\;\left(t\right)}}\right)\\ + & \frac{\partial S}{\partial \sigma_{\alpha }\;\left(t\right)}\sum_{\gamma }K_{\alpha \gamma }J_{\alpha \gamma }\;\left({\bar{c}}_{\gamma \beta }+\frac{{\bar{a}}_{\beta }}{N_{\beta }}\delta_{\gamma \beta }\right)\underset{=O\;\left(\frac{h_{\text{ext}}}{\sqrt{K}}\right)}{\underset{︸}{\delta \sigma_{\alpha }\;\left(t\right)}}\\ + & \left.S\;\left({\bar{\mu }}_{\alpha },{\bar{\sigma }}_{\alpha }\right)K_{\alpha \beta }J_{\alpha \beta }\frac{\left(1-2\bar{m}{}_{\beta }\right)}{N_{\beta }}\delta m_{\beta }\;\left(t\right)\right\}+\left\{\alpha ↔\beta \right\} \end{array}$$ (30)

Before finally solving for *δc* (*t*), we want to justify the assumption $\delta c=O\left(\frac{\delta m}{N}\right)$, which we needed already in the beginning to determine *δm* (*t*), by a short calculation. We insert Eq (22) into Eq (30) and switch to matrix notation for brevity, which yields

$$\begin{array}{cc} & \tau \frac{d}{dt}\delta c\;\left(t\right)+\left\{\left(1-\bar{S}KJ\right)\delta c\;\left(t\right)\right\}+{\left\{...\right\}}^{T}\\ = & \left\{\frac{\partial S}{\partial \mu }KJ\;\left(\bar{c}+\frac{\bar{a}}{N}\right)\;\left(h_{\text{ext}}\;\text{sin}(\omega t)+KJ\delta m\;\left(t\right)\right)+\underset{=\frac{\partial S}{\partial \mu }\;\left(\frac{\Theta -\mu }{\sigma }-\frac{\sigma }{\Theta -\mu }\right)}{\underset{︸}{\frac{\partial S}{\partial \sigma }}}KJ\;\left(\bar{c}+\frac{\bar{a}}{N}\right)\right.\\ & \left.\times \;\left(\frac{KJ^{2}\;\left(1-2\bar{m}\right)}{2\bar{\sigma }}\delta m\;\left(t\right)+\frac{KJ}{2\sigma }\delta c\;\left(t\right)\;{\left(KJ\right)}^{T}\right)+\bar{S}KJ\frac{1-2\bar{m}}{N}\delta m\;\left(t\right)\right\}+{\left\{...\right\}}^{T}. \end{array}$$ (31)

We can rewrite the left hand side in order to recognize the parts, which are identical to the right hand side of Eq (23), i.e. the ODE for *δm* (*t*) without the neglect of *δσ*, which gives

$$\begin{array}{l} \begin{array}{c} \{\frac{\partial S}{\partial \mu }KJ\;\left(\bar{c}+\frac{\bar{a}}{N}\right)\underset{=\;\left(\tau \frac{\partial }{\partial t}\delta m\;\left(t\right)+\delta m\;\left(t\right)\right)/S}{\underset{︸}{\left(\left(h_{\text{ext}}\text{ sin}\left(\omega t\right)+KJ\delta m\;\left(t\right)\right)+\frac{\Theta -\mu }{\sigma }\;\left(\frac{KJ^{2}\;\left(1-2\bar{m}\right)}{2\bar{\sigma }}\delta m\;\left(t\right)+\frac{KJ}{2\sigma }\delta c\;\left(t\right)\;{\left(KJ\right)}^{T}\right)\right)}} \end{array}\\ -\underset{=\frac{1}{\bar{\sigma }}\frac{\Theta -\bar{\mu }}{\bar{\sigma }}\bar{S}}{\underset{︸}{\frac{\partial S}{\partial \mu }}}\frac{\sigma }{\Theta -\mu }KJ\;\left(\bar{c}+\frac{\bar{a}}{N}\right)\;\left(\frac{KJ^{2}\;\left(1-2\bar{m}\right)}{2\bar{\sigma }}\delta m\;\left(t\right)+\frac{KJ}{2\sigma }\delta c\;\left(t\right)\;{\left(KJ\right)}^{T}\right)\\ +\bar{S}KJ\frac{1-2\bar{m}}{N}\delta m\;\left(t\right)\}+{\left\{\ldots \right\}}^{T}. \end{array}$$ (32)

Bringing the *δc*-terms on the left hand side finally yields

$$\begin{array}{l} \begin{array}{c} \tau \frac{d}{dt}\delta c\;\left(t\right)+\left\{\left(1-\bar{S}KJ\right)\delta c\;\left(t\right)+\underset{=O\;\left(\bar{S}KJ\frac{K}{N}\frac{KJ^{2}}{\sigma^{2}}\delta c\;\left(t\right)\right)=O\;\left(\bar{S}KJ\frac{K}{N}\delta c\;\left(t\right)\right)}{\underset{︸}{\frac{1}{\bar{\sigma }}\bar{S}KJ\;\left(\bar{c}+\frac{\bar{a}}{N}\right)\frac{KJ}{2\sigma }\delta c\;\left(t\right)\;{\left(KJ\right)}^{T}}}\right\}+{\left\{\ldots \right\}}^{T} \end{array}\\ \begin{array}{c} =\{\underset{=O\left(\bar{S}KJ\frac{1}{N}\delta m\left(t\right)\right)}{\underset{︸}{\underset{=\frac{1}{\sigma }\frac{\Theta -\bar{\mu }}{\bar{\sigma }}}{\underset{︸}{\frac{\frac{\partial S}{\partial \mu }}{S}}}KJ\left(\bar{c}+\frac{\bar{a}}{N}\right)\left(\tau \frac{\partial }{\partial t}\delta m_{\alpha }\left(t\right)+\delta m_{\alpha }\left(t\right)\right)}} \end{array}\\ \begin{array}{c} -\underset{=O\;\left(\bar{S}KJ\frac{1}{N}\delta m\;\left(t\right)\right)}{\underset{︸}{\frac{1}{\bar{\sigma }}\bar{S}KJ\;\left(\bar{c}+\frac{\bar{a}}{N}\right)\frac{KJ^{2}\;\left(1-2\bar{m}\right)}{2\bar{\sigma }}\delta m\;\left(t\right)}}+\bar{S}KJ\frac{1-2\bar{m}}{N}\delta m\;\left(t\right)\}+{\left\{\ldots \right\}}^{T}. \end{array} \end{array}$$ (33)

We have therefore shown that—independent of the scaling of the synaptic weights *J*—the relation $\delta c=O\left(\frac{\delta m}{N}\right)$ holds not only for the zero-mode, i.e. for the stationary case, but also for the time-dependent part. Note that for our actual calculation of *δm*, we have neglected its dependence on *δσ*, as it is one order $\sqrt{K}$ smaller than the *δμ*-contribution. However, this is not true for *δc* because of the cancellation of the two contributions to *δμ*. Inserting the rhs of the ODE Eq (24) actually used to determine *δm* and shifting the *δc*-contribution of *δσ* back to the other side, we arrive at

$$\begin{array}{cc} & \tau \frac{d}{dt}\delta c\;\left(t\right)+\left\{\left(1-\bar{S}KJ\right)\delta c\;\left(t\right)\right\}+{\left\{...\right\}}^{T}\\ = & \left\{\frac{\frac{\partial S}{\partial \mu }}{S}KJ\;\left(\bar{c}+\frac{\bar{a}}{N}\right)\;\left(\tau \frac{\partial }{\partial t}\delta m_{\alpha }\;\left(t\right)+\delta m_{\alpha }\;\left(t\right)\right)\right.\\ & -\frac{1}{\bar{\sigma }}\;\left(1-{\left(\frac{\mu -\Theta }{\sigma }\right)}^{2}\right)\bar{S}KJ\;\left(\bar{c}+\frac{\bar{a}}{N}\right)\;\left(\frac{KJ^{2}\;\left(1-2\bar{m}\right)}{2\bar{\sigma }}\delta m\;\left(t\right)+\frac{KJ}{2\sigma }\delta c\;\left(t\right)\;{\left(KJ\right)}^{T}\right)\\ & \left.+\bar{S}KJ\frac{1-2\bar{m}}{N}\delta m\;\left(t\right)\right\}+{\left\{...\right\}}^{T}. \end{array}$$ (34)

We want to compare the contribution from *δμ* in the second line of Eq (34) with the contribution from *δσ* in the third line. As pointed out above, they scale in the same way with the system size *N*, given that we do not rescale the driving frequency with *N*. Therefore, its contribution stays equally important if we enlarge the network. We neglect it anyway, which can be justified by comparing the decisive part of the prefactors of the *δσ* and the *δμ*-parts (the remaining parts are of the same order of magnitude):

$$\begin{array}{c} \bar{\sigma }\frac{\frac{\partial S}{\partial \mu }}{S}=\frac{\Theta -\bar{\mu }}{\bar{\sigma }}=\sqrt{2}{\text{erfc}}^{-1}\left(2\bar{m}\right)\overset{\text{for}\;\text{input}\;\text{fluct.}\;\text{not}\;\text{too}\;\text{small}}{\gg }\frac{1}{\bar{\sigma }}\exp\left(-{\text{erfc}}^{-1}{\left(2\bar{m}\right)}^{2}\right)=\bar{S}. \end{array}$$

This inequality is fulfilled for the three settings used in this work, whereas the first term is one or two orders of magnitude larger than the second. Especially, this inequality can always be fulfilled if the externally generated noise level is high. Therefore, even if the neglect of the *δσ*-contribution to *δc* cannot be justified by the standard mean-field argument that it decays faster with the system size than other terms, it is applicable because the input fluctuations are large enough—for all system sizes. This largely simplifies the calculations because the ODE for *δc* can be solved by transforming into the eigensystem of *W*, which would not be possible after including the more involved term emerging from *δσ*. Taking into account the neglected term would require to reformulate the problem as an equation for the vector (*δc*_EE_, *δc*_EI_, ‥), which would be much less intuitive. Furthermore, there is an indirect argument for high frequencies that does rely on the system size: The *ω*-dependence of the absolute value of the maxima of *δm* and *δc* scales with the eigenvalues of *W*, which scale with $\sqrt{K}$. Thus, changing the system size *N* in first order just stretches the *ω*-axis. Therefore, the “interesting” frequencies do scale with *N*, which leads to the dominance of the derivative term in the second line of Eq (34) over the *δσ*-term. Note that the observation from Eq (32) that

$$\begin{array}{cc} & \underset{=O\;\left(h_{\text{ext}}\right)}{\underset{︸}{{\left(KJ\;\left(1+O\;\left(\frac{1}{\sqrt{K}}\right)\right)\delta m\right)}^{\text{diag}}}}+\underset{=O\;\left(h_{\text{ext}}\right)}{\underset{︸}{h_{\text{ext}}\text{ sin }\left(\omega t\right)}} \end{array}$$ (35)

$$\begin{array}{c} ={\left({\bar{S}}^{\text{diag}}\right)}^{-1}\;\left(\tau \frac{\partial }{\partial t}\delta m+\delta m\right)+O\;\left(\sqrt{\left|K\right|}\left|\delta m\right|\right)=O\;\left(\sqrt{\left|K\right|}\left|\delta m\right|\right)=O\;\left(\frac{h_{\text{ext}}}{\sqrt{\left|K\right|}}\right) \end{array}$$ (36)

is a direct consequence of the recurrent drive being effectively inhibitory (for other networks, the expansion around the stationary point would not make sense): Any of the two terms in the susceptibility terms are of order $\sqrt{K}$ bigger than their sum. Furthermore, we see from Eq (33) that the sum of the susceptibility terms is of the same order of magnitude with respect to its dependence on *K* (or, equivalently, the connection probabilities and the system size) as the term coming from the time modulation of the variances (modulated-autocovariances-drive).

We define

$$\begin{array}{ccc} T_{\alpha \beta } & ≔ & K_{\alpha \beta }J_{\alpha \beta }\\ V_{\alpha \beta } & ≔ & \frac{\Theta -{\bar{\mu }}_{\alpha }}{{\left({\bar{\sigma }}_{\alpha }\right)}^{2}}S\;\left({\bar{\mu }}_{\alpha },{\bar{\sigma }}_{\alpha }\right)K_{\alpha \beta }J_{\alpha \beta }, \end{array}$$ (37)

and

$$\begin{array}{ccc} N_{\alpha \beta }^{\text{diag}} & = & \delta_{\alpha \beta }N_{\alpha }\\ {\bar{m}}_{\alpha \beta }^{\text{diag}} & = & \delta_{\alpha \beta }{\bar{m}}_{\alpha }\\ {\bar{a}}_{\alpha \beta }^{\text{diag}} & = & \delta_{\alpha \beta }{\bar{a}}_{\alpha }\\ \delta m_{\alpha \beta }^{\text{diag}}\left(t\right) & = & \delta_{\alpha \beta }\delta m_{\alpha }\left(t\right)\\ {\left(T\delta m\left(t\right)\right)}_{\alpha \beta }^{\text{diag}} & = & \delta_{\alpha \beta }\sum_{\gamma }T_{\alpha \gamma }\delta m_{\gamma }\left(t\right), \end{array}$$ (38)

in order to end up with the index-free version Eq (10). The first two inhomogeneities, the susceptibility terms introduced in the main part (“Results“) reflect the nonlinearity of the gain-function.

With *U* given in Eq (25), we multiply Eq (30) from the left by *U*^−1^ and from the right by (*U*^−1^)^*T*^ to get (cf. [13, 33])

$$\begin{array}{ccc} & & \tau \frac{d}{dt}\underset{≔\tilde{\delta c}}{\underset{︸}{U^{-1}\delta c\;\left(t\right)\;{\left(U^{-1}\right)}^{T}}}\\ & = & \left\{\left(-1+\right.\right.\underset{=\Lambda }{\underset{︸}{U^{-1}WU}}\left.\right)\underset{≔\tilde{\delta c\;\left(t\right)}}{\underset{︸}{U^{-1}\delta c\;\left(t\right)\;{\left(U^{-1}\right)}^{T}}}\\ & + & U^{-1}\;\left({\left(T\delta m\;\left(t\right)\right)}^{\text{diag}}+h_{\text{ext}}\text{ sin }\left(\omega t\right)\right)V\;\left(\bar{c}+\frac{1}{N^{\text{diag}}}{\bar{a}}^{\text{diag}}\right)\;{\left(U^{-1}\right)}^{T}\\ & + & U^{-1}W\;\left(1-2{\bar{m}}^{\text{diag}}\right)\frac{1}{N^{\text{diag}}}\left.\delta m{\left(t\right)}^{\text{diag}}\;{\left(U^{-1}\right)}^{T}\right\}\\ & + & {\left\{...\right\}}^{T}. \end{array}$$

We are only interested in the cyclostationary statistics, so we can ignore again the transient state making the ansatz $\tilde{\delta c_{\alpha \beta }^{\text{inhom}}}=\tilde{C_{\alpha \beta }^{1}}e^{i\omega t}$. Inserting this ansatz and transforming back into the original system, we get

$$\begin{array}{l} \tilde{C_{\alpha \beta }^{1}}=h_{\text{ext}}\frac{-i\tau \omega +2-\left(\lambda_{\alpha }+\lambda_{\beta }\right)}{{\left(\tau \omega \right)}^{2}+{\left(2-\left(\lambda_{\alpha }+\lambda_{\beta }\right)\right)}^{2}}\\ \;[\sum_{\gamma ,\delta ,\theta ,\phi ,\eta }U_{\alpha \eta }^{-1}U_{\beta \delta }^{-1}T_{\eta \theta }U_{\theta \phi }{\left(U^{-1}S\right)}_{\phi }\frac{-i\tau \omega +1-\lambda_{\phi }}{{\left(\tau \omega \right)}^{2}+{\left(1-\lambda_{\phi }\right)}^{2}}V_{\eta ,\gamma }{\left(\bar{c}+\frac{1}{N^{\text{diag}}}{\bar{a}}^{\text{diag}}\right)}_{\gamma \delta }\\ \;+\sum_{\gamma ,\delta ,\epsilon }U_{\alpha \epsilon }^{-1}U_{\beta \delta }^{-1}V_{\epsilon \gamma }{\left(\bar{c}+\frac{1}{N^{\text{diag}}}{\bar{a}}^{\text{diag}}\right)}_{\gamma \delta }\\ \;+\sum_{\theta ,\phi ,\gamma }U_{\alpha \gamma }^{-1}U_{\beta \theta }^{-1}W_{\gamma \theta }\left(1-2{\bar{m}}_{\theta }^{\text{diag}}\right)\frac{1}{N_{\theta }}U_{\theta \phi }{\left(U^{-1}S\right)}_{\phi }\frac{-i\tau \omega +1-\lambda_{\phi }}{{\left(\tau \omega \right)}^{2}+{\left(1-\lambda_{\phi }\right)}^{2}}] \end{array}$$ (39)

Together with Eq (9), this is the main result of this section.

## Discussion

The present work offers an extension of the well-known binary neuronal network model beyond the stationary case [6, 13, 27, 28, 33]. We here describe the influence of a sinusoidally modulated input on the mean activities and the covariances to study the statistics of recurrently generated network activity in an oscillatory regime, ubiquitously observed in cortical activity [18].

Comparing with the results of the simulation of the binary network with NEST [35, 36] and the numerical solution of the full mean-field ODE, we are able to show that linear perturbation theory is sufficient to explain the most important effects occurring due to sinusoidal drive. This enables us to understand the mechanisms by the help of analytical expressions and furthermore we can predict the network response to any time-dependent perturbation with existing Fourier representation by decomposing the perturbing input into its Fourier components.

We find that the amplitude of the modulation of the mean activity is of the order $h_{\text{ext}}/{\left({\left(1-\lambda_{\alpha }\right)}^{2}+{\left(\tau \omega \right)}^{2}\right)}^{\frac{1}{2}}$, where λ_*α*_, *α* ∈ {*E*, *I*} are the eigenvalues of the effective connectivity matrix *W*, i.e. the input is filtered by a first order low-pass filter and the amplitude of the modulation decays like ∝ *ω*^−1^ for large frequencies. This finding is in line with earlier work on the network susceptibility [27, esp. section V].

The qualitatively new result here is the identification of two distinct mechanisms by which the covariances *δc* are modulated in time. First, covariances are driven by the direct modulation of the susceptibility *S* due to the time-dependent external input and by the recurrent input from the local network. Second, time-modulated variances, analogous to their role in the stationary setting [13], drive the pairwise covariances.

Our setup is the minimal network model, in which these effects can be observed—minimal in the sense that we would lose these properties if we further simplified the model: The presence of a nonlinearity in the neuronal dynamics, here assumed to be a threshold-like activation function, is required for the modulation of covariances by the time-dependent change of the effective gain. In a linear rate model [10, 46] this effect would be absent, because mean activities and covariances then become independent.

The second mechanism relies on the binary nature of neuronal signal transmission: the variance *a*(*t*) of the binary neuronal signal is, at each point in time, completely determined by its mean *m*(*t*). This very dependence provides the second mechanism by which the temporally modulated mean activity causes time-dependent covariances, because all fluctuations and therefore all covariances are driven by the variance *a*(*t*).

Rate models have successfully been used to explain the smallness of pairwise covariances [6] by negative feedback [10]. A crucial difference is that their state is continuous, rather than binary. As a consequence, the above-mentioned fluctuations present due to the discrete nature of the neuronal signal transmission need to be added artificially: The pairwise statistics of spiking or binary networks are equivalent to the statistics of rate models with additive white noise [46]. To obtain qualitative or even quantitative agreement of time-dependent covariances between spiking or binary networks and rate models, the variance of this additive noise needs to be chosen such that its variance is a function of the mean activity and its time derivative.

The direct modulation of the susceptibility *S* due to the time-dependent external input leads to a contribution to the covariances with first order low-pass filter characteristics that dominates the modulated covariances at large frequencies. For small—and probably biologically realistic—frequencies (typically the LFP shows oscillations in the *β*-range around 20 Hz), however, the modulation of the susceptibility by the local input from the network leads to an equally important additional modulation of the susceptibility. The intrinsic fluctuations of the network activity are moreover driven by the time-dependent modulation of the variance, which is a function of the mean activity as well. Because the mean activity follows the external drive in a low-pass filtered manner, the latter two contributions hence exhibit a second order low-pass-filter characteristics. These contributions are therefore important at the small frequencies we are interested in here.

The two terms modulating the susceptibility, by the direct input and by the feedback of the mean activity through the network, have opposite signs in balanced networks. In addition they have different frequency dependencies. In networks in which the linearized connectivity has only real eigenvalues, these two properties together lead to their summed absolute value having a maximum. Whether or not the total modulation of the covariance shows resonant behavior, however, depends also on the third term that stems from the modulated variances. We find that in purely inhibitory networks, the resonance peak is typically overshadowed by the latter term. This is because inhibitory feedback leads to negative average covariances [13], which we show here reduce the driving force for the two resonant contributions. In balanced E-I networks, the driving force is not reduced, so the resonant contribution can become dominant.

For the biologically motivated parameters used in the last setting studied here, the effective coupling matrix *W* has complex eigenvalues which cause resonant mean activities. If the inhomogeneity was independent of the driving frequency, *δc* would have resonant modes with frequency *f*_res_ and 2*f*_res_. Due to the mixing of the different modes and by the frequency dependence of the inhomogeneity driving the modulation of covariances, these modes determine only the ballpark for the location of the resonance in the covariance. Especially the resonances are not sharp enough so that each of them is visible in any combination of the modes. Different behavior is expected near the critical point where ℜ(λ) ≲ 1.

For predictions of experimental results, however, a more careful choice of reasonable biological parameters would be necessary. In particular, the external drive should be gauged such that the modulations of the mean activities are in the experimentally observed range. Still, our setup shows that the theory presented here works in the biologically plausible parameter range.

The goal of extracting fundamental mechanisms of time-dependent covariances guides the here presented choice of the level of detail of our model. Earlier works [6, 28, 29] showed that our setup without sinusoidal drive is sufficient to qualitatively reproduce and explain phenomena observed in vivo, like high variability of neuronal activity and small covariances. The latter point can be explained in binary networks by the suppression of fluctuations by inhibitory feedback, which is a general mechanism also applicable to other neuron models [10] and even finds application outside neuroscience, for example in electrical engineering [47]. The high variability observed in binary networks can be explained by the network being in the balanced state, that robustly emerges in the presence of negative feedback [29, 30]. In this state, the mean excitatory and inhibitory synaptic inputs cancel so far that the summed input to a neuron fluctuates around its threshold. This explanation holds also for other types of model networks and also for biological neural networks [48]. We have seen here that the operation in the balanced state, at low frequencies, gives rise to a partial cancellation of the modulation of covariances.

Our assumption of a network of homogeneously connected binary neurons implements the general feature of neuronal networks that every neuron receives input from a macroscopic number of other neurons, letting the impact of a single synaptic afferent on the activation of a cell be small and the summed input be distributed close to Gaussian: For uncorrelated incoming activity, the ratio between the fluctuations caused by a single input and the fluctuations of the total input is $N^{-\frac{1}{2}}$, independent of how synapses scale with *N*. However, the input to a neuron is actually not independent, but weakly correlated, with covariances decaying at least as fast as *N*^−1^ [6, 29]. Therefore this additional contribution to the fluctuations also decays like $N^{-\frac{1}{2}}$. The Gaussian approximation of the synaptic input relies crucially on these properties. Dahmen et al. [39] investigated third order cumulants, the next order of non-Gaussian corrections to this approximation. They found that the approximation has a small error even down to small networks of about 500 neurons and 50 synaptic inputs per neuron. These estimates hold as long as all synaptic weights are of equal size. For distributed synaptic amplitudes, in particular those following a wide or heavy-tailed distributions (e.g. [49, 50], reviewed in [51]), we expect the simple mean-field approximation applied here to require corrections due to the strong effect of single synapses.

The generic feature of neuronal dynamics, the threshold-like nonlinearity that determines the activation of a neuron, is shared by the binary, the leaky integrate-and-fire and, approximately, also the Hodgkin-Huxley model neuron. An important approximation entering our theory is the linearity of the dynamic response with respect to the perturbation. We estimate the validity of our theory by comparison to direct simulations. To estimate the breakdown of this approximation we compare the linear response to the first non-linear correction. We observe that the second order harmonics in the considered range of parameters remains as small as about 10 percent of the first harmonics. The quadratic contribution to the transfer properties of the neurons stems from the curvature of the effective gain function *φ* (Eq (17)). The linear portion of this gain function, in turn, is controlled by the amplitude *σ* of the synaptic noise. One therefore expects a breakdown of the linear approximation as soon as the temporal modulation of the mean input is of the order of this amplitude. Fig D in S1 Text shows that with the parameters *h*_ext_ = 1 and *σ*_exc,inh_ ≈ 10, used in the plots Figs 5 and 6 and Fig. B and Fig. C in S1 Text, the linear approximation is good, whereas in Fig 7, we used *h*_ext_ = 6, for which the linear perturbation theory already begins to break down. The latter figure is mainly supposed to give an intuitive impression.

A generic property that is shared by nearly all neuron models is the characteristic duration *τ* during which the activity of a sending cell affects the downstream neuron. For the binary neuron model, this time scale is identical to the mean interval *τ* between updates, because, once active, a neuron will stay active until the next update. It most certainly deactivates at that point, because we here consider low activity states prevalent in cortex [1]. In the leaky integrate-and-fire model the exponentially decaying membrane voltage with time constant *τ* is qualitatively similar: it sustains the effect that an input has on the output for this time scale. As a consequence, neurons transmit their input in a low-pass filtered manner to their output. This feature persists for more realistic spiking models, as shown for the leaky integrate-and-fire model [52, 53], the exponential integrate-and-fire model [52, 53], and the quadratic integrate-and-fire model [54]. We therefore expect that the qualitative properties reported here will carry over to these models.

A possible application of the framework developed in this paper is a quantitative comparison of the neuronal activity in the model network to the analysis of data measured in cortex [26]. Detecting the occurrence of so called Unitary Events (UE, [55–57], see also Sec. I in S1 Text), the authors observed that the simultaneous activation of neurons above the level expected for independence is locked to certain phases of the LFP. They hypothesized that the reason for this observation is the activation of cell assemblies. The results presented here show that the correlated activation of pairs of neurons is modulated by a sinusoidal drive even in a completely unstructured random network. In consequence, the locking of pairwise events to the cycle of the LFP is more pronounced for correlated events than for single spikes. Future work needs to quantitatively compare experimental data to the results from the model presented here. The closed form expressions for the modulations of the mean activities and covariances enable such an approach and the effective study of the dependence on the model parameters. A quantitative comparison needs to convert mean activities and pairwise covariances for binary neurons into the probability to measure a unitary event, interpreting the binary neuron states as binned spike trains. Preliminary results indicate that already the homogeneous network presented in this work can show some features described in [26]. In Sec. I in S1 Text, we apply the Unitary Event analysis to our setting. The presented methods will be helpful to analyze the modulation of synchrony in the presence of cell assemblies [58] in the model. This can be done by enhancing the connection probability among groups of excitatory neurons, similar as in [59] and will yield a more realistic model, which captures also nonlinear effects in the perturbation. Technically this extension amounts to the introduction of additional populations and the change of the connectivity matrix to reflect that these populations represent cell assemblies.

The relation of spiking activity to mesoscopic measures, such as the LFP, is still an open question. These population measures of neuronal activity naturally depend on the statistics of the microscopic activity they are composed of. Pairwise covariances, the focus of the current work, in particular tend to dominate the variance of any mesoscopic signal of summed activity: The contribution of covariances grows quadratically in the number of components, the contribution of variances only linearly [60, Box 2][10, eq. (1)][21, eq. (1),(2)]. Under the assumption that the LFP mainly reflects the input to a local recurrent network [21, 24], we have shown here that these two signals—spikes and LFPs—are intimately related; not only does the afferent oscillatory drive trivially modulate the propensity to produce spikes, their firing rate, but also the joint statistics of pairs of neurons by the three distinct pathways exposed in the present analysis. Forward modeling studies have shown that the spatial reach of the LFP critically depends on covariances, with elevated covariances leading to larger reach [21]. In this light our work shows that a local piece of neuronal tissue driven by a source of coherent oscillations will more effectively contribute to the local field potential itself: not only the spiking rate is modulated accordingly, but also the covariances are increased and decreased in a periodic manner, further amplifying the modulation of the generated local field potential and temporally modulating the spatial reach of the signal.

Functional consequences of the findings presented here deduce from the hypothesis that communication channels in cortex may effectively be multiplexed by the selective excitation of different areas with coherent oscillations [61, 62]. The presented analysis exposes that oscillatory drive to a local piece of cortex alone already effectively enhances coherent firing beyond the level expected based on the assumption of independence. If synchronous activity is employed as a dimension to represent information, it is hence tightly entangled with time-dependent changes of the mean activity. A similar conclusion was drawn from the observation that covariance transmission in feed-forward networks is monotonously increasing with firing rate [4, 5]. Any information-carrying modulation of synchronous activity must hence go beyond the here investigated effects, which can be regarded the baseline given by the non-stationary activity in networks without function. Since the mechanisms we have exposed only depend on generic features of cortical tissue—networks of non-linear neurons, connectivity with strong convergence and divergence, and dynamic stabilization by inhibition—the time-dependent entanglement of mean activity and covariances qualitatively exists in any network with these properties. In this view, our analysis can help to distinguish the level of time-modulated covariances in neural tissues that are surprising, and are therefore candidates to be attributed to function, from those that need to be expected in networks due to their generic properties.

## Supporting information

S1 Text Appendix. In this text, we show how to apply the UE-analysis to a model network of binary neurons choosing the parameters from Table 1. We also present the derivation of the ODEs for the first two moments, we discuss the different possibilities to define a spike in a binary network and show how to handle the complex phase jump induced by the usage of the sine-function as a perturbation. Furthermore, we include the plots of the II- and EI-component of the covariances for the parameters of Table 1 and a plot of the mean activities and the EE-covariance for varying perturbation strength *h*_ext_ for the same parameters.
(PDF)
