# Supplementary material for: Locking of correlated neural activity to ongoing oscillations
Source: PLoS Comput Biol. 2017 Jun 12;13(6):e1005534. doi: 10.1371/journal.pcbi.1005534 (PMC5484611; doi:10.1371/journal.pcbi.1005534)
Supplement: S1 Text — In this text, we show how to apply the UE-analysis to a model network of binary neurons choosing the parameters from Table 1. We also present the derivation of the ODEs for the first two moments, we discuss the different possibilities to define a spike in a binary network and show how to handle the complex phase jump induced by the usage of the sine-function as a perturbation. Furthermore, we include the plots of the II- and EI-component of the covariances for the parameters of Table 1 and a plot of the mean activities and the EE-covariance for varying perturbation strength hext for the same parameters. (PDF) [file pcbi.1005534.s001.pdf]

Appendix of "Locking of correlated neural activity to ongoing oscillations", PLoS CB 2017

Tobias Kuehn[1] and Moritz Helias[1,2]

[1] *Institute of Neuroscience and Medicine (INM-6) and Institute for Advanced Simulation (IAS-6) and JARA BRAIN Institute I, Jülich Research Centre, Jülich, Germany*
[2] *Department of Physics, Faculty 1, RWTH Aachen University, Aachen, Germany*
(Dated: May 21, 2017)

In this text, we show how to apply the UE-analysis to a model network of binary neurons choosing the parameters from Table 1. We also present the derivation of the ODEs for the first two moments, we discuss the different possibilities to define a spike in a binary network and show how to handle the complex phase jump induced by the usage of the sine-function as a perturbation. Furthermore, we include the plots of the II- and EI-component of the covariances for the parameters of Table 1 and a plot of the mean activities and the EE-covariance for varying perturbation strength h_{ext} for the same parameters.

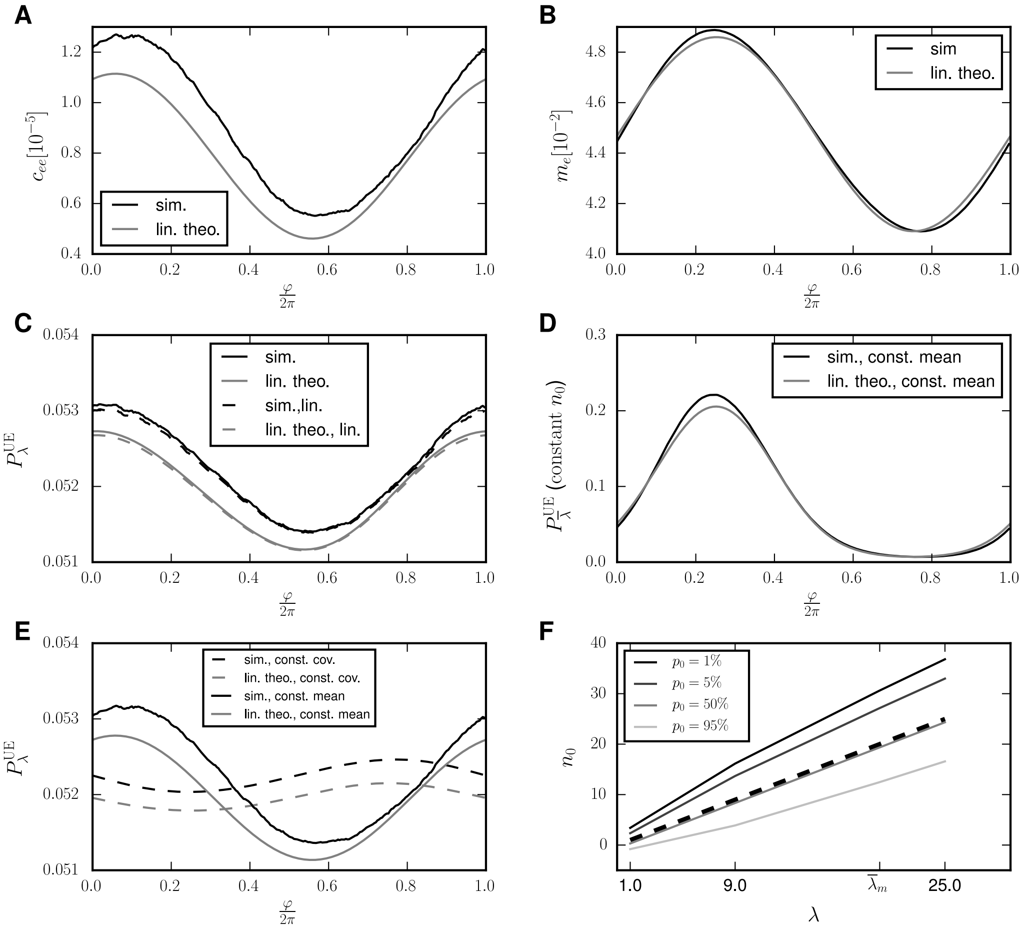

Figure A. Temporal modulation of Unitary Events. Covariance $c(\varphi)$ (A) mean activity $m(\varphi)$ (B) as functions of the phase φ of the LFP cycle. C Probability $P_\lambda^{\mathrm{UE}}(\varphi)$ for the appearance of a significant number of Unitary Events as a function of the phase of the oscillation. Solid curves show the exact expression App-Eq (2), dashed curves the corresponding approximation to linear order in c, App-Eq (3). D $P_{\overline{\lambda}}^{\mathrm{UE}}(\varphi)$ for a constant n_0, adjusted to the time-averaged mean activity. E $P_\lambda^{\mathrm{UE}}(\varphi)$ for constant mean activity and time-dependent covariance (solid curves) and vice versa (dashed curves). For the plots in A-E, black curves always represent simulation results and gray curves $N_{\mathrm{bin}} = 10000$ and $p_0 = 5$ and $f = 160\,\mathrm{Hz}$. The parameters for the network simulation are given in Table 1. F Dependence of the lowest number required for a UE n_0 on the average rate λ for different significance level p_0, $\overline{\lambda}_m$ indicates the stationary part of the rate used for the plots A-E.

I. APPLICATION OF THE UNITARY EVENT ANALYSIS TO CORRELATED NETWORK ACTIVITY

We will give a concise, but self-contained description of the main idea of a Unitary Event analysis and its application to our setup. The observation of at least n_0 simultaneous spikes in a time series of N_{bin} bins is called Unitary Event (UE, [1–3]). We are therefore interested in the the time-dependence of the probability $p_{ij}(t)$ that the pair of neurons i and j fires together at time t, which causes a time-dependence of the covariance $c(t)$. Concretely, because the appearance of a spike is a binary event, the probability of the joint firing is identical to the second moment $p_{ij}(t) = \langle n_i(t) n_j(t) \rangle$ [see also 4, eq. 22] which, in turn, can be expressed as

$$p_{ij}(t) = \langle n_i(t) n_j(t) \rangle = c_{ij}(t) + m_i(t)\, m_j(t). \tag{1}$$

The covariance therefore enters this probability in an additive manner. The significance test of the Unitary Event analysis, depending on the momentary rate, aims to eliminate the contribution of the trivial second term. One therefore expects that the modulation of the covariance influences also the probability to observe a Unitary Event.

Concretely, one assumes that the number of joint firing events is Poisson distributed, therefore the probability to observe a UE is given by

$$P_\lambda^{\mathrm{UE}}(n_0) = \sum_{n>n_0} e^{-\lambda_m} \frac{\lambda_m^n}{n!},$$

where $\lambda = m^2 N_{\mathrm{bin}}$ for a uncorrelated system with mean activity m and the number of bins N_{bin} and n_0 is chosen minimal such that $P_{\mathrm{UE}}(n_0) < p_0$ for a given significance level p_0, in [5] for example, $p_0 = 0.05$. In our setup, m changes continuously in time, thus the limitation $n_0 \in \mathbb{N}$ is unfavorable. Therefore, we replace the cumulative Poisson distribution by a cumulative distribution yielding the same values on $\mathbb{N}$, but being defined on $\mathbb{R}$. That is fulfilled by

$$f(\lambda, n_0) := P_\lambda^{\mathrm{UE}}(n_0) = \frac{\gamma(n_0+1, \lambda)}{\Gamma(n_0+1)} =: \frac{\int_0^\lambda t^{n_0} e^{-t} dt}{\int_0^\infty t^{n_0} e^{-t} dt}.$$

$\Gamma(n_0)$ and $\gamma(n_0)$ are the Gamma- and the incomplete Gamma-function, respectively. This correspondence follows from the third last equality in App-Eq (4) and $\Gamma(n_0+1) = n_0!\ \forall n_0 \in \mathbb{N}$. Here f is monotonous in n_0, therefore we can define a function $f^{-1}(\lambda, p_0)$ via

$$f\left(\lambda, f^{-1}(\lambda, p_0)\right) = p_0.$$

Now, we want to determine the probability to observe a UE in a correlated system, that is $\lambda = \lambda_m + \lambda_c =: \left(m^2 + c\right) N_{\mathrm{bin}}$ in case that n_0 is determined assuming a uncorrelated system. For the systems described in this work, being in the balanced state, we can safely assume that the covariance c is small and therefore enters in P_λ^{UE} only in linear order:

$$\begin{aligned} & f\left(\lambda_m + \lambda_c, f^{-1}(\lambda_m, p_0)\right) && (2) \\ =& f\left(\lambda_m, f^{-1}(\lambda_m, p_0)\right) + \partial_1 f\left(\lambda_m, f^{-1}(\lambda_m, p_0)\right)\lambda_c + \mathcal{O}\left(\lambda_c^2\right) \\ =& p_0 + \partial_1 f\left(\lambda_m, f^{-1}(\lambda_m, p_0)\right)\lambda_c + \mathcal{O}\left(\lambda_c^2\right) && (3) \end{aligned}$$

($\partial_1 f$ means the derivative of f with respect to its first argument). The following computation

$$\begin{aligned} & P_\lambda^{\mathrm{UE}}(n_0 > X \geq n_0 - 1) \\ =& P_\lambda^{\mathrm{UE}}(X \geq n_0 - 1) - P_\lambda^{\mathrm{UE}}(X \geq n_0) \\ =& \frac{\int_0^\lambda t^{n_0-1} e^{-t} dt}{\int_0^\infty t^{n_0-1} e^{-t} dt} - \frac{\int_0^\lambda t^{n_0} e^{-t} dt}{\int_0^\infty t^{n_0} e^{-t} dt} \\ \overset{\text{P.I.}}{=}& \frac{\lambda^{n_0} e^{-\lambda}}{\int_0^\infty t^{n_0} e^{-t} dt} = \frac{\partial}{\partial \lambda} P_\lambda(X \geq n_0) && (4) \\ =& \partial_1 f\left(\lambda_m, f^{-1}(\lambda_m, p_0)\right) \end{aligned}$$

leads to an illustrative interpretation of App-Eq (3): For $c > 0$, λ_c is the number of additional joint firing events that one expects due to the positive covariance and $\partial_1 f$ is the probability to observe one joint firing event less than the minimal number n_0, that is required for a UE in the uncorrelated system. Therefore, in this approximation, the required number of joint firing events for the classification as UE stays the same, only the probability to observe this many joint firing events is elevated by $P_\lambda^{\mathrm{UE}}(s > X \geq s-1)\lambda_c$.

If we neglect any time-dependence and determine just a constant n_0 according to the time-averaged mean activity, P_λ^{UE} is misestimated for our network (Figure A, D). Determining n_0, we therefore have to consider the time-dependence of m. To this end, we can assume that the time-varying part is small compared to the stationary part, that is

$$\begin{aligned} \lambda &= \overline{\lambda}_m + \delta\lambda_m(t) + \overline{\lambda}_c + \delta\lambda_c(t) \\ &= \left(\overline{m}^2 + 2\overline{m}\delta m(t) + \overline{c} + \delta c(t) + \mathcal{O}(\delta m)^2\right) T. \end{aligned}$$

The qualitative effect of a time-dependent mean activity (which causes a instantaneous shift in n_0) in a network with constant positive covariance can now be seen by the following argument: Assume that one could instantaneously adjust the covariance such that $n_0(t) = \lambda_m(t) + \lambda_c(t)$, that is, we construct a system that produces *on average* the number of joint firing events required *at the minimum* to be classified as a UE. Like that, the surprise of an observer

knowing this covariance is always on the same level. Following this construction, a small deviation in $\lambda_m(t)$ around some stationary value $\overline{\lambda}_m$ will force us to also shift $\lambda_c(t)$ a bit according to

$$\delta\lambda_c(t) = \left(\frac{\partial n}{\partial \lambda} - 1\right)\delta\lambda_m(t).$$

From Figure AF, we can read off that $\frac{\partial n}{\partial \lambda} > 1$ for small p_0. We therefore need $\delta\lambda_c(t)$ to modulate in phase with $\delta\lambda_m(t)$ to keep the surprise constant. In turn keeping λ_c constant will lower the probability for a UE, if λ_m is raised. This argument explains that the UE-probability assuming constant, nonzero covariance modulates in antiphase with $m(t)$, as shown by the dashed curves in panel E. The solid lines in the same panel show that for a constant mean activity, P_λ^{UE} modulates proportional to $\lambda_c(t)$ (or $c(t)$, respectively), as expected from App-Eq (3). The actual UE-probability, shown in C, is a superposition of both effects. The comparison to the linear approximation, shown by the dashed curves, reveals that neglecting higher order contributions of λ_c is indeed appropriate. As expected from App-Eq (1), the probability of Unitary Events is elevated because the covariance is positive. As the time-dependent part of the covariance itself is dominated by the linear response, we overall get a dominating first harmonic in the modulation of $P_{\mathrm{UE}}(t)$. As a consequence, we cannot obtain a locking that is strongly localized at a certain phase of the LFP, in contrast to the experimental observation (cf. Fig 6 of [5]).

A quantitative examination would require a Taylor expansion of $\partial_1 f\left(\lambda_m, f^{-1}(\lambda_m, p_0)\right)$ in $\delta\lambda_m(t)$, which gives two contributions with different signs. The first one is positive and arises because $\delta\lambda_m > 0$ causes a rise in $P_\lambda^{\mathrm{UE}}(n_0 > X \geq n_0 - 1)$ for n_0 kept constant, the second is negative and comes up because $\delta\lambda_n > 0$ causes a positive shift in n_0 which lowers $P_\lambda^{\mathrm{UE}}(n_0 > X \geq n_0 - 1)$ for λ_m kept constant. Numerical checks seem to show that the last contribution is dominant for the interesting parameter range leading to $\frac{d}{d\lambda_m}\partial_1 f\left(\lambda_m, f^{-1}(\lambda_m, p_0)\right) < 0$, as expected because of the qualitative argument given before.

II. SOME THEORETICAL AND TECHNICAL DETAILS

A. Derivation of the moment equations using the Master equation

For completeness, we here derive the differential equations equations for the first and second moments Equation (15), following previous work [4, 6–9].

We multiply the Master equation by n_k or $n_l n_k$ respectively and get

$$\begin{aligned}
\tau\frac{d}{dt}\langle n_k\rangle(t) &= \sum_{\boldsymbol{n}\in\{0,1\}^N}\frac{d}{dt}p(\boldsymbol{n},t)\,n_k = \sum_{\boldsymbol{n}\backslash n_k} n_l\phi_k(\boldsymbol{n}\backslash n_k,t)\\
&= \sum_{\boldsymbol{n}\in\{0,1\}^N} n_k\sum(2n_i-1)\,\phi_i(\boldsymbol{n}\backslash n_i,t)\\
&= \sum_{\boldsymbol{n}\in\{0,1\}^N}\left(n_k\phi(\boldsymbol{n}\backslash n_k,t) + \underbrace{n_k\sum_{i\neq k}^{N}(2n_i-1)\,\phi_i(\boldsymbol{n}\backslash n_i,t)}_{=0}\right)\\
&= \sum_{\boldsymbol{n}\backslash n_k}\left[-p(\boldsymbol{n}_{k+},t) + \left(p(\boldsymbol{n}_{k-},t)\,F_k(\boldsymbol{n}_{k-}) + p(\boldsymbol{n}_{k+},t)\,F_k(\boldsymbol{n}_{k+})\right)\right]\\
&= -\langle n_k\rangle(t) + \langle F_k(t)\rangle
\end{aligned}$$

and

$$\begin{aligned}
\frac{d}{dt}\langle n_k(t)\, n_l(t)\rangle &= \sum_{\boldsymbol{n}\in\{0,1\}^N} \frac{d}{dt} p(\boldsymbol{n},t)\, n_k n_l \\
&= \sum_{\boldsymbol{n}\in\{0,1\}^N} n_k n_l \sum_{i=1}^{N} (2n_i - 1)\,\phi_i(\boldsymbol{n}\backslash n_i, t) \\
&= \sum_{\boldsymbol{n}\in\{0,1\}^N} \Big(n_k n_l \phi_k(\boldsymbol{n}\backslash n_k, t) + n_l n_k \phi_l(\boldsymbol{n}\backslash n_l, t) \\
&\quad + \underbrace{n_k n_l \sum_{i\neq k,l}^{N} (2n_i - 1)\,\phi_i(\boldsymbol{n}\backslash n_i, t)}_{=0} \Big) \\
&= \sum_{\boldsymbol{n}\backslash n_k} n_l \phi_k(\boldsymbol{n}\backslash n_k, t) + k \leftrightarrow l \\
&= \sum_{\boldsymbol{n}\backslash n_k} \left[-n_l p(\boldsymbol{n}_{k+}, t) + n_l \left(p(\boldsymbol{n}_{k-}, t) F_k(\boldsymbol{n}_{k-}) + p(\boldsymbol{n}_{k+}, t) F_k(\boldsymbol{n}_{k+}) \right) \right] \\
&\quad + k \leftrightarrow l \\
&= \left\{ -\langle n_k(t)\, n_l(t)\rangle + \langle n_l(t)\, F_k(t)\rangle \right\} + \{k \leftrightarrow l\}\,.
\end{aligned}$$

B. Different definitions for a spiking event of a binary neuron

In [10], van Vreeswijk et al. identify the transition $0 \to 1$ with a spike, which leads to the equation $\nu_\alpha = \frac{m_\alpha(1-m_\alpha)}{\tau}$ for the firing rate. We think, however, that this identification is inappropriate in our case, because the $0 \to 1$-transition for a binary neuron has a different meaning than a spike for a spiking neuron. In our opinion, it is decisive, for which fraction of time a spiking neuron affects the downstream neurons. If it spikes with frequency ν_α and the membrane potential decays with the time constant τ, this fraction is given by $\tau\nu_\alpha$. This can be interpreted as the mean activity of a spiking neuron, which leads to the definition of the firing rate of a binary neuron $\nu_\alpha = \frac{m_\alpha}{\tau}$ in section Two populations with inhomogeneous connections. In other words: If we want to identify a spiking event for a binary neuron, we will have to count the $1 \to 1$-transition as spike as well. For small mean activities, however, the difference is small anyway.

C. Extracting the correct phase from complex solutions

Notice that there are a few subtleties to keep in mind when a discrete Fourier transform is applied to δm_α. The (in both senses) real-valued solution of the ODE is

$$\begin{aligned}
\delta m_\alpha &= \Im\left(M_\alpha^1 e^{i\omega_0 t}\right) = \Im\left(\left|M_\alpha^1\right| e^{i\left(\arg\left(M_\alpha^1\right)+\omega_0 t\right)}\right) = \left|M_\alpha^1\right| \sin\left(\arg\left(M_\alpha^1\right) + \omega_0 t\right) \\
&= \left|M_\alpha^1\right| \left(\sin\left(\arg\left(M_\alpha^1\right)\right)\cos(\omega_0 t) + \cos\left(\arg\left(M_\alpha^1\right)\right)\sin(\omega_0 t)\right).
\end{aligned}$$

For clarity, we here named the driving frequency ω_0. Therefore, if we calculate the Fourier transform (in a distributional sense), we get

$$\begin{aligned}
&\mathcal{F}\left[\delta m_\alpha\right](\omega = \omega_0) \\
&= \left|M_\alpha^1\right| \left(\sin\left(\arg\left(M_\alpha^1\right)\right) \frac{\delta_{\omega_0} + \delta_{-\omega_0}}{2} + \cos\left(\arg\left(M_\alpha^1\right)\right) \frac{\delta_{\omega_0} - \delta_{-\omega_0}}{2i}\right) \\
&= \frac{\left|M_\alpha^1\right|}{2} \left(\delta_{\omega_0}\left(\sin\left(\arg\left(M_\alpha^1\right)\right) - i\cos\left(\arg\left(M_\alpha^1\right)\right)\right) + \delta_{-\omega_0}\left(\sin\left(\arg\left(M_\alpha^1\right)\right) + i\cos\left(\arg\left(M_\alpha^1\right)\right)\right)\right)
\end{aligned}$$

Thus, we get

$$|\mathcal{F}[\delta m_\alpha](\omega_0)| = |\mathcal{F}[\delta m_\alpha](-\omega_0)| = \frac{|M_\alpha^1|}{2}$$

and, because we take the imaginary part of the complex solution which leads to a $\frac{\pi}{2}$-phase shift compared to the complex phase

$$\arg(\mathcal{F}[\delta m_\alpha](\omega_0)) = \begin{cases} \arg(M_\alpha^1) + \frac{3\pi}{2}, & \text{for } \arg(M_\alpha^1) \in [-\pi, -\frac{\pi}{2}] \\ \arg(M_\alpha^1) - \frac{\pi}{2}, & \text{for } \arg(M_\alpha^1) \in (-\frac{\pi}{2}, \pi). \end{cases}$$

III. COMPARISON OF SIMULATION AND THEORY OF THE EI AND II-COVARIANCES AND VALIDATION OF THE LINEAR PERTURBATION THEORY

For completeness, we include here the plots showing the dependence of the covariances between inhibitory and inhibitory and excitatory on the driving frequency for the third network setup of the main text. In Figure D, we show that the linear perturbation theory breaks down if the perturbation is of the same order as the input fluctuations.

IV. ACKNOWLEDGEMENTS

The authors would like to thank PierGianLuca Porta Mana for his great support and Michael Denker, Sonja Grün and the whole INM-6 for fruitful discussions. All network simulations carried out with NEST (http://www.nest-simulator.org).

[1] S. Grün, A. Aertsen, M. Abeles, and G. Gerstein, in *The Self-Organizing Brain. From Growth Cones to Functional Networks*, edited by J. van Pelt, M. Corner, H. Uylings, M. P. van Veen, and A. van Ooyen (Amsterdam, Netherlands Inst Brain Res, Royal Netherl Acad Sci, Grad School Neurosci, Amsterdam, 1993), pp. 94–95.
[2] S. Grün, M. Diesmann, and A. Aertsen, Neural Comput. **14**, 43 (2002).
[3] S. Grün, M. Diesmann, and A. Aertsen, Neural Comput. **14**, 81 (2002).
[4] R. Glauber, J. Math. Phys. **4**, 294 (1963).
[5] M. Denker, S. Roux, H. Lindén, M. Diesmann, A. Riehle, and S. Grün, Cereb. Cortex **21**, 2681 (2011).
[6] A. Renart, J. De La Rocha, P. Bartho, L. Hollender, N. Parga, A. Reyes, and K. D. Harris, Science **327**, 587 (2010).
[7] M. Helias, T. Tetzlaff, and M. Diesmann, PLOS Comput. Biol. **10**, e1003428 (2014).
[8] I. Ginzburg and H. Sompolinsky, Phys. Rev. E **50**, 3171 (1994).
[9] M. A. Buice, J. D. Cowan, and C. C. Chow, Neural Comput. **22**, 377 (2010).
[10] C. Van Vreeswijk and H. Sompolinsky, Neural Comput. **10**, 1321 (1998).

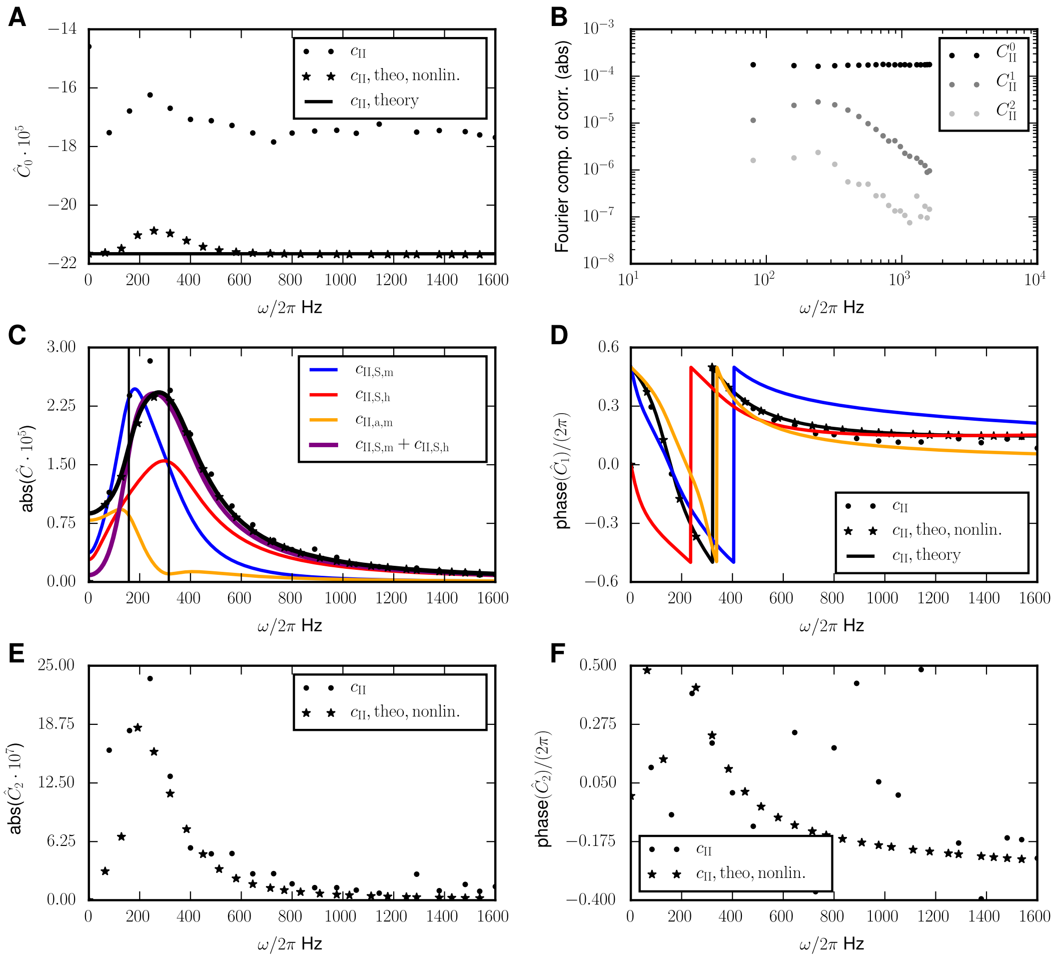

Figure B. **Driven E-I network with biologically inspired parameters: II-Covariance.** Response of the inh.-inh.-part of the covariance to a perturbation with frequency ω in the Fourier space. **A** Zeroth order (time independent part) of the covariance. **B** Absolut value of the first three Fourier components of the c_{II}-covariances in loglog-scale. **C** Absolute value of the first order of the time-dependent part of the covariance. **D** Phase angle in relation to the driving signal. **E** and **F** analogous to **C** and **D** for the second Fourier modes. Solid lines indicate the linear theory Equation (41), stars the results of the numerical solved full mean-field theory Equation (5) and Equation (6) and dots those of the direct simulation of the full network. Numerical results obtained by the same methods and identical parameters as in Figure 5.

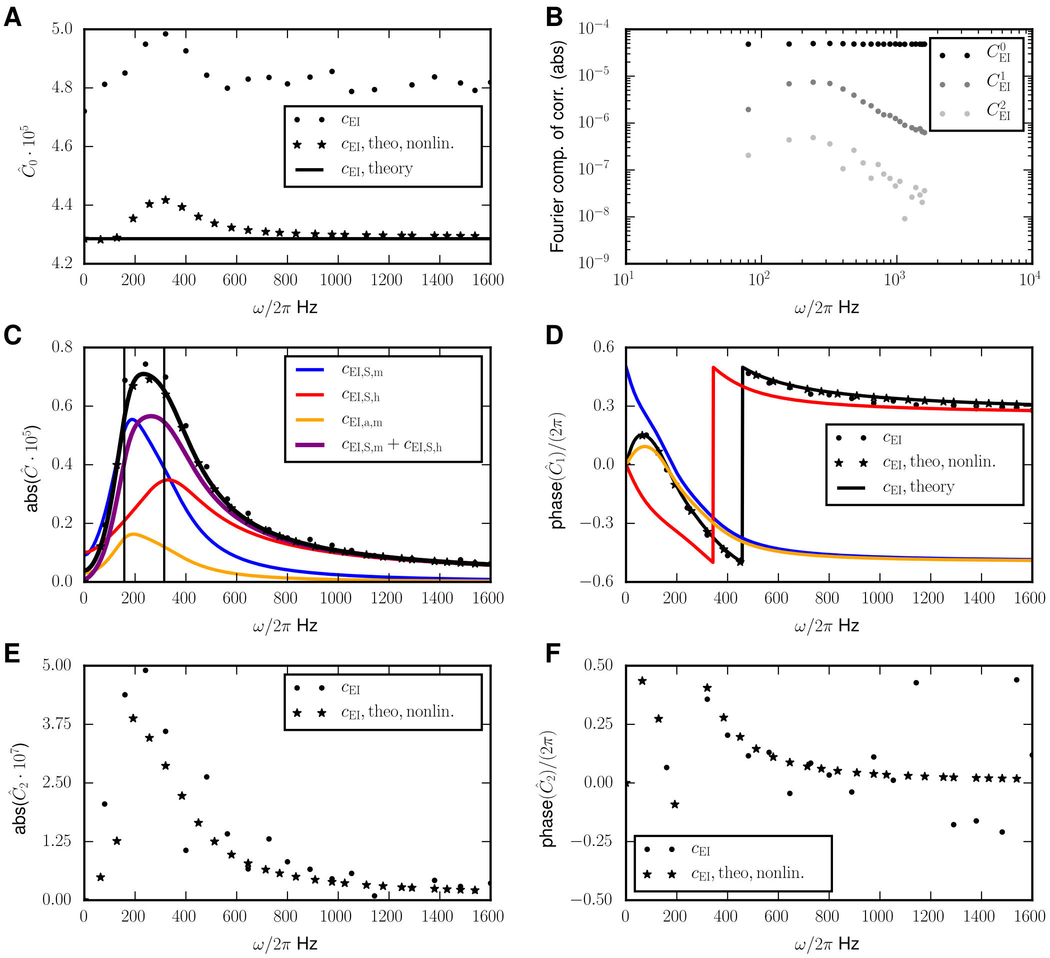

Figure C. **Driven E-I network with biologically inspired parameters: EI-Covariance.** Response of the exc.-inh.-part of the covariance to a perturbation with frequency ω in the Fourier space. **A** Zeroth order (time independent part) of the covariance. **B** Absolut value of the first three Fourier components of the c_{EI}-covariances in loglog-scale. **C** Absolute value of the first order of the time-dependent part of the covariance. **D** Phase angle in relation to the driving signal. **E** and **F** analogous to **C** and **D** for the second Fourier modes. Solid lines indicate the linear theory Equation (41), stars the results of the numerical solved full mean-field theory Equation (5) and Equation (6) and dots those of the direct simulation of the full network. Numerical results obtained by the same methods and identical parameters as in Figure 5.

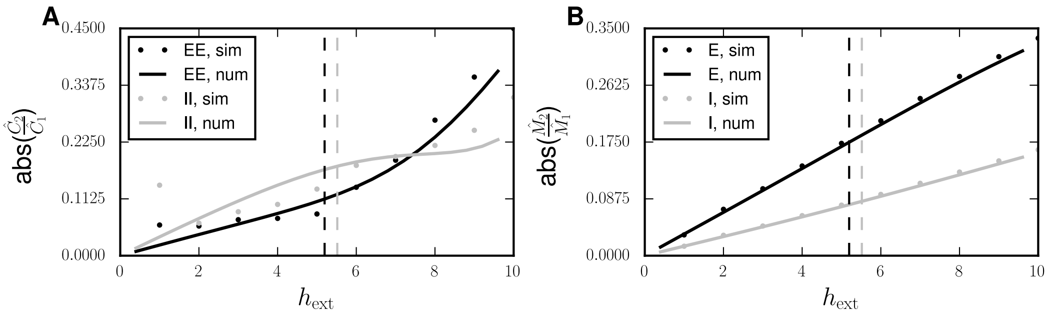

Figure D. **Driven E-I network with biologically inspired parameters: Dependence of the covariance and the mean activity on h_{ext}.** Ratio of the second to the first Fourier component in a system subject to a perturbation with frequency $\omega = 20 \cdot 2\pi$Hz. **A** Covariance between excitatory and between inhibitory neurons. **B** Mean activity of the excitatory and of the inhibitory population. The vertical dotted lines indicate $\sigma_{\mathrm{exc.}}/2$ (black) and $\sigma_{\mathrm{inh.}}/2$ (lightgray). Solid lines indicate the results of the numerical solved full mean-field theory Equation (5) and Equation (6) and dots those of the direct simulation of the full network. Numerical results obtained by the same methods and with the same parameters as in Figure 3.
